# What Are the Key Gut Microbiota Involved in Neurological Diseases? A Systematic Review

**DOI:** 10.3390/ijms232213665

**Published:** 2022-11-08

**Authors:** Bruno Bonnechère, Najaf Amin, Cornelia van Duijn

**Affiliations:** 1 REVAL Rehabilitation Research Center, Faculty of Rehabilitation Sciences, Hasselt University, 3590 Diepenbeek, Belgium; 2 Nuffield Department of Population Health, University of Oxford, Oxford OX3 7LF, UK

**Keywords:** gut microbiome, Parkinson’s disease, Alzheimer’s disease, stroke, multiple sclerosis, atrophic lateral sclerosis

## Abstract

There is a growing body of evidence highlighting there are significant changes in the gut microbiota composition and relative abundance in various neurological disorders. We performed a systematic review of the different microbiota altered in a wide range of neurological disorders (Alzheimer’s disease (AD), Parkinson’s disease (PD), multiple sclerosis (MS), amyotrophic lateral sclerosis, and stroke). Fifty-two studies were included representing 5496 patients. At the genus level, the most frequently involved microbiota are Akkermansia, Faecalibacterium, and Prevotella. The overlap between the pathologies was strongest for MS and PD, sharing eight genera (Akkermansia, Butyricicoccus, Bifidobacterium, Coprococcus, Dorea, Faecalibacterium, Parabacteroides, and Prevotella) and PD and stroke, sharing six genera (Enterococcus, Faecalibacterium, Lactobacillus, Parabacteroides, Prevotella, and Roseburia). The identification signatures overlapping for AD, PD, and MS raise the question of whether these reflect a common etiology or rather common consequence of these diseases. The interpretation is hampered by the low number and low power for AD, ALS, and stroke with ample opportunity for false positive and false negative findings.

## 1. Introduction

**Figure 1:**
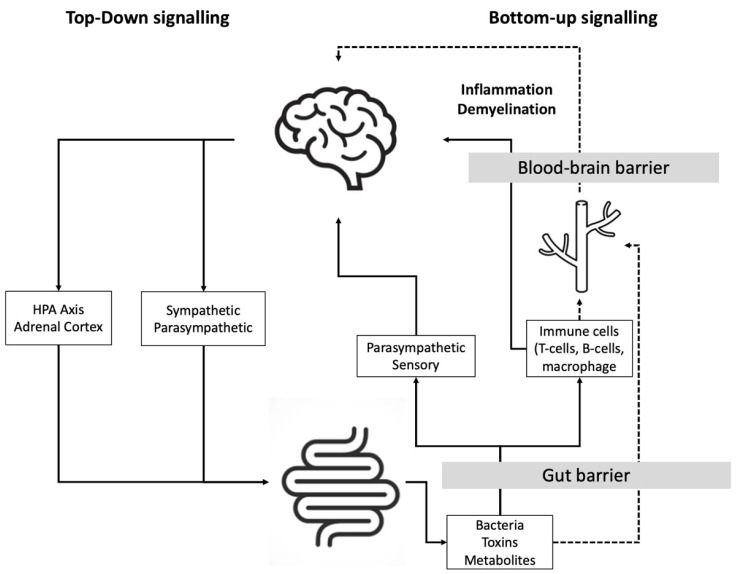
Diagram depicting the communication between the gut and the brain. Multiple pathways, including the autonomic nervous system, enteric nervous system, hypothalamic–pituitary–adrenal (HPA), immune pathways, endocrine pathways, and neural pathways, all have a strong influence on this bidirectional relationship, adapted from [36,37,38].

The role and importance of the bidirectional communications between the brain and the gut—also referred to as the brain–gut axis [1]—in the pathogenesis of various central nervous disorders [2] is receiving increasing interest in recent years and is, probably, one of the most promising areas of research [3,4,5]. The gut microbiome is composed of a vast number of microorganisms: about 1000 bacterial species and 7000 bacterial strains have been identified representing a total of 1013–1014 different microorganisms in the gut [6,7]. The gut–brain axis is a bidirectional communication axis involving the intestinal microbiome, the intestinal barrier, intestinal inflammation, and the intestinal/systemic/brain immune systems, among other components [8] (see the different communications and pathways in Figure 1). The gut–brain axis contributes to normal central nervous system function and pathology [9,10].

Modifications of the compositions of the gut microbiota have been identified in numerous pathologies and the different potential mechanisms of action have been described in a significant number of narrative reviews mostly focusing on Alzheimer’s disease (AD) [11,12,13,14,15,16], Parkinson’s disease (PD) [17,18,19,20,21,22,23], and multiple sclerosis (MS) [24,25,26,27]. Changes in the gut microbiota have also been found in stroke [28,29] and amyotrophic lateral sclerosis (ALS) [30,31].

A main field of research seeks to determine whether modification of the gut microbiota can decrease the risk of developing diseases or improve the health of the patients in AD [32], PD [33], MS [34], and ALS [35].

Clinical studies have been fueled by the findings in transgenic animal models for AD and PD, which strongly support the interplay between gut microbiota and AD and PD in the brain and identified the gut microbiome as a key determinant of the risk of dementia and a potential target for preventive interventions. In AD transgenic mice, changes in the gut microbiome have been shown to alter amyloid deposition in the brain [39,40]. PD and other synucleinopathies are characterized by the aggregation of α-synuclein protein (αSyn) overexpression in mice, which results in motor dysfunction. It has been shown that gut microbiota influence motor deficits, microglial activation, and αSyn pathology and appear to be a necessary part in pathogenesis [41]. Antibiotic treatment ameliorates pathophysiology in adult animals, whereas microbial re-colonization promotes it, suggesting that postnatal signaling between the gut and the brain modulates disease [42].

In humans, recent evidence suggests that probiotic treatment can improve cognition of patients with mild cognitive impairment by improving the regulation of gut microbiota [43]. Modification of the gut microbiota can also be obtained by modifying the diet—particularly promising results have been found in various neurological conditions using a ketogenetic diet [44]. Despite a growing body of evidence [45], quantitative evidence about the various microbiota involved in neurological diseases is lacking. Many unanswered questions remain: from a physiopathological perspective, it is important to determine if some gut microbiota are consistently linked to a specific disorder across studies to understand their mechanism of action in a specific disease, while from a research perspective it is crucial to evaluate whether findings are consistent. It is also of interest to determine if the associations and modifications are associated with multiple neurological diseases. This may suggest either a common pathogenesis or a common progression of the disease, i.e., neurological pathology may lead to changes in the gut microbiome or vice-versa. Either pathway may be of interest. A causal pathway may open opportunities for prevention and intervention if the neurological disease leads to changes in the gut microbiome. It is, therefore, important to identify the changes in microbiota in the different pathologies, and to determine the direction of these changes in order to enhance management through personalized treatment and precision medicine [46].

In this review, we summarize the findings of the studies of microbiome association with neurodegenerative disorders including AD, PD, and ALS. We further compare these to the findings of microbiome studies on MS, which is a neuroinflammatory disease, and stroke, which often co-occurs with AD and PD.

## 2. Methods

### 2.1. Search Strategy

**Figure 2:**
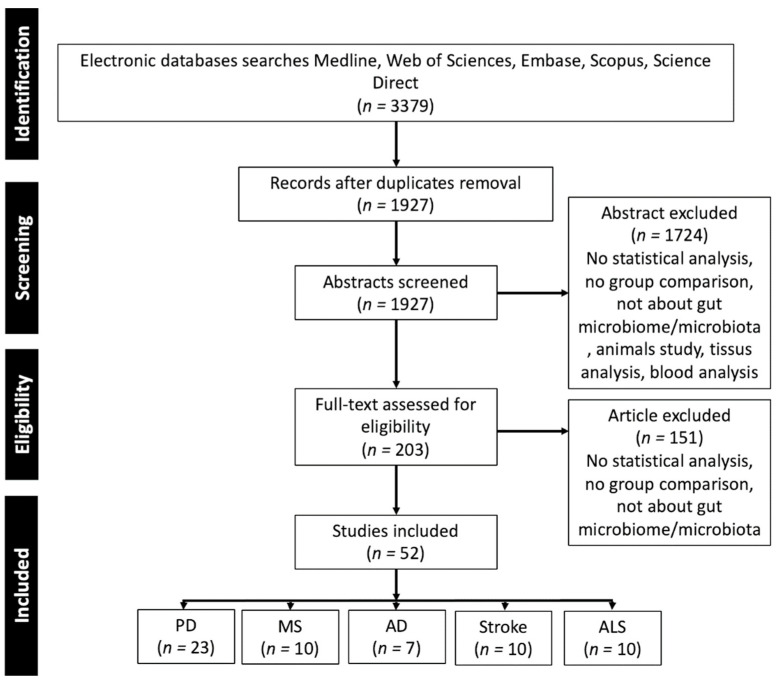
PRISMA flow diagram of the studies selection.

To review the relation of the microbiome in the gut to neurological disorders in the human population, we performed a literature review that included articles published prior to 1 January 2022 with a combination of terms “gut”, “microbiome”, “stool”, “fecal” and the different pathologies “Alzheimer’s disease”, “Parkinson’s disease”, “Multiple Sclerosis”, “Stroke”, “Amyotrophic lateral sclerosis”. Additional relevant articles were sought through a manual bibliography search. Inclusion criteria were: human research, focus on AD, PD, MS, AMS, and stroke that makes a comparison between patients and healthy controls, focus on gut microbiota quantified from stool samples, and articles published in English in peer-reviewed journals. A flow diagram of study selection is presented in Figure 2.

### 2.2. Data Extraction

From the included studies the following parameters were extracted: the country where the experiments have been carried out, general characteristics of the patients and controls (e.g., age, BMI, sex ratio), and microbiome analysis method. We then listed the different taxon in which relative abundance has been found statistically significantly different between patients and healthy controls and indicated the direction: relative abundance increased or decreased in the various diseases under study.

### 2.3. Statistical Analysis

We determined whether findings were consistent across studies of a single disorder based on statistical significance and the direction of association for the studies that showed significant results. If two studies were found to show the same direction of the relationship (e.g., microbiota taxus was found more often or less often in patients in both studies), findings were classified as consistent. As most studies were small, one expects heterogeneity across studies due to random fluctuation. We therefore also considered findings for which the consistency was more than 50% as consistent. We present the phylogenetic trees of the microbiota that have been consistently found in the literature for the different pathologies using the GraPhIAn package in Python. General information about the included studies and baseline data on patients were summarized in graphics. To compare the different microbiota involved in the studied pathologies we used Venn diagrams using the VennDiagram package in R [47].

## 3. Results

### 3.1. Review of the Gut Microbiome in PD, AD, MS, Stroke, and ALS

In total, 52 studies were included in the final analysis, representing 3177 patients and 2319 controls; the repartition of the patients within the different pathologies is presented in Supplementary Figure S1. The figure shows that PD is the most frequently studied disorder, comprising 54% of all patients and 44% of all studies. As the gut microbiome is known to depend on the environment and diet, which differ across societies and thus across studies, the distribution of the studies per country is presented in Supplementary Figure S2. It is of note that most of the studies were conducted in China (*n* = 16, 31%), Northern and Eastern Europe (*n* = 18, 35% for Germany, Italy, Finland, Luxembourg, and the Netherlands), and in the USA (*n* = 10, 19%).

The characteristics for for PD patients in Supplementary Table S1, for AD patients in Supplementary Table S2, for MS patients in Supplementary Table S3, for stroke patients in Supplementary Table S4, and in Supplementary Table S5 for ALS patients.

**Figure 3:**
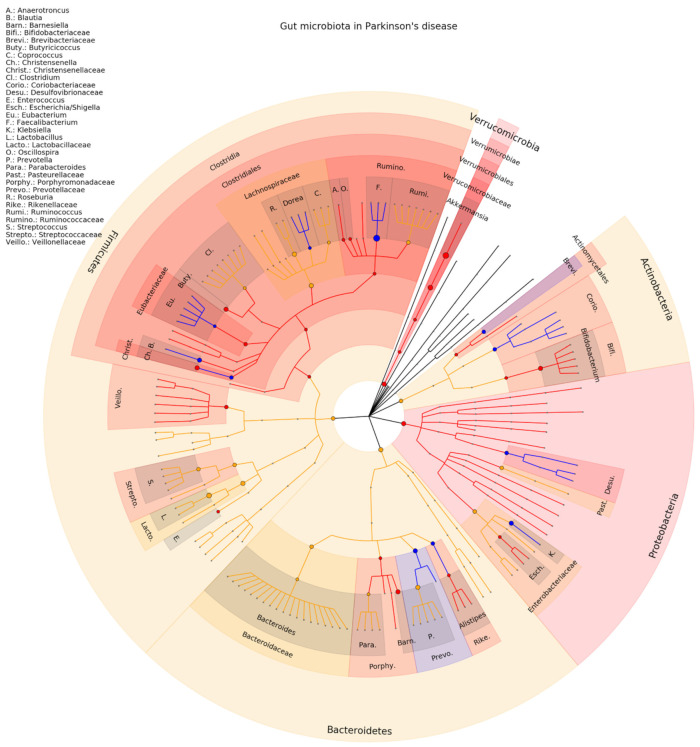
Phylogenetic distributions of the microbiota involved in Parkinson’s. Orange indicates inconsistent results, blue indicates a decrease in relative abundance, and red indicates an increase in relative abundance. These results are the summary of consistent findings of the included studies [48,49,50,51,52,53,54,55,56,57,58,59,60,61,62,63,64,65,66,67,68,69,70].

We found a total of 1709 PD patients and 1224 healthy controls [48,49,50,51,52,53,54,55,56,57,58,59,60,61,62,63,64,65,66,67,68,69,70]. At the genus level, 60 gut microbiota were identified in at least one study, 16 (25%) associations to PD were consistent in terms of significance and direction (Akkermansia, Alistipes, Anaerotroncus, Barnesiellaceae, Bifidobacterium, Blautia, Butyricicoccus, Christensenella, Dorea, Enterococcus, Escherichia/Shigella, Eubacterium, Faecalibacterium, Fusinibacter, Klebsiella, Oscilospira, Parabacteroides). The association between Akkermansia and PD is replicated in nine studies, that of Bifidobacterium and Faecalibacterium in seven studies. For seven microbiota (Bacteroides, Coprococcus, Lactobacillus, Parabacteroides, Prevotella, Roseburia, Streptococcus) at least two studies were found in the same direction, while for two (Clostridium, Ruminococcus) there were four studies suggesting opposite direction. A phylogenetic tree representing the results is presented in Figure 3, while the complete results of the included studies are presented in Supplementary Table S6.

**Figure 4:**
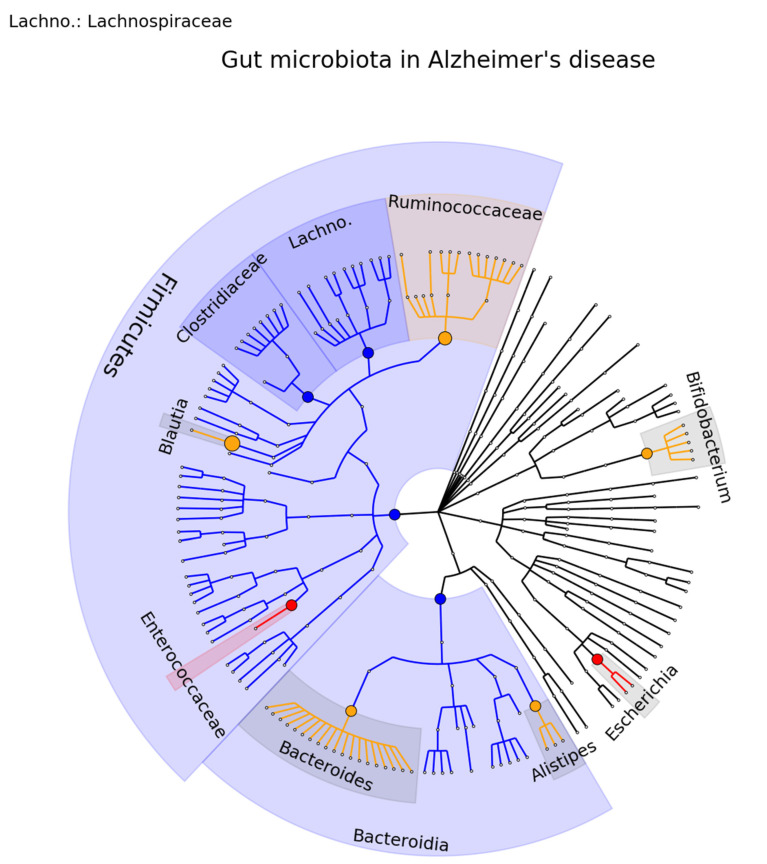
Phylogenetic distributions of the microbiota involved in Alzheimer’s disease. Orange indicates inconsistent results, blue indicates a decrease in relative abundance, and red indicates an increase in relative abundance. These results are the summary of consistent findings of the included studies [71,72,73,74,75,76,77].

For AD, a smaller number of studies were found (*n* = 7), with each study comprising only a limited number of patients. Due to the small sample size of individual studies, nine gut microbiota were reported to be associated with AD in only a single study. Integrating the data of 258 AD patients and 216 healthy controls [71,72,73,74,75,76,77], we find at the family level consistent findings for the Clostridiaceae, Enterococcaceae, and Lachnospiraceae. However, at the genus level, only one association (6%) was found to be consistent (Escherichia/Shigella). For four (25%) associations (Alistipes, Bacteroides, Bifidobacterium, Blautia) at least two studies were found in the same direction. A phylogenetic tree representing the results is presented in Figure 4, and the complete results are presented in Supplementary Table S7.

**Figure 5:**
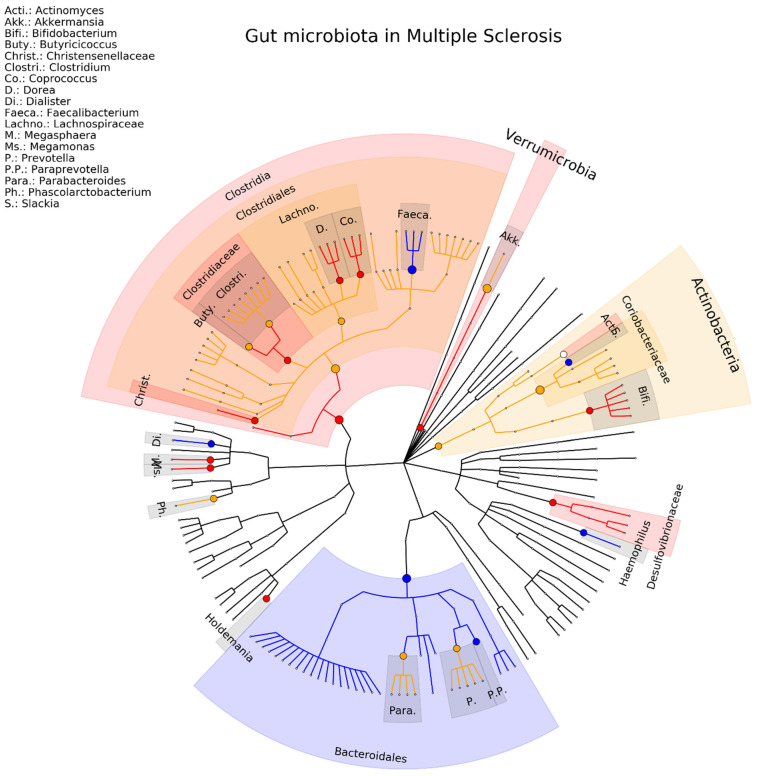
Phylogenetic distributions of the microbiota involved in multiple sclerosis. Orange indicates inconsistent results, blue indicates a decrease in relative abundance, and red indicates an increase in relative abundance. These results are the summary of consistent findings of the included studies [78,79,80,81,82,83,84,85,86,87].

We found ten studies on MS, involving a total of 307 MS patients and 311 healthy controls [78,79,80,81,82,83,84,85,86,87]. Despite the small number of patients and controls, there was a relatively high consistency seen at the genus level involving 11 (18%) associations (Actinomyces, Akkermansia, Bifidobacterium, Coprococcus, Dialister, Dorea, Faecalibacterium, Haemophilus, Megasphaera, Paraprevotella, Slackia) and only six (10%) associations in the opposite direction (Butyricicoccus, Clostridium, Gemmiger, Parabacteroides, Phascolarctobacterium, Prevotella) (see Figure 5 and Supplementary Table S8).

**Figure 6:**
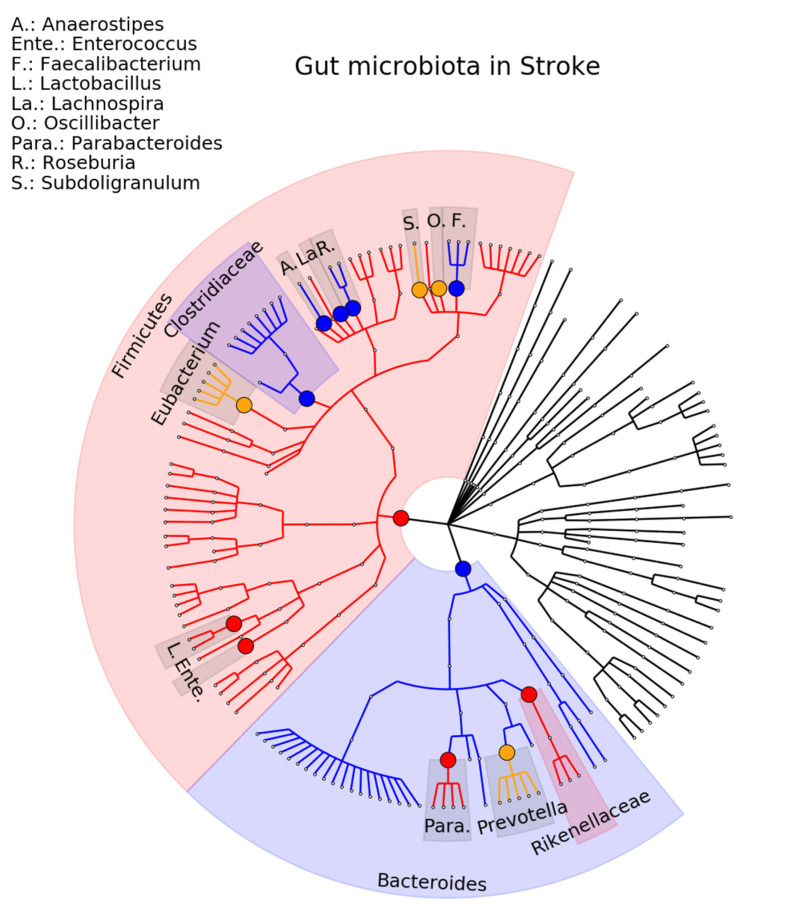
Phylogenetic distributions of the microbiota involved in stroke. Orange indicates inconsistent results, blue indicates a decrease in relative abundance, and red indicates an increase in relative abundance. These results are the summary of consistent findings of the included studies [88,89,90,91,92,93].

Six studies on stroke were included in the review, involving a total of 744 stroke patients and 403 healthy controls [88,89,90,91,92,93]. At the genus level, 42 associations were seen in one study, seven (13%) associations to stroke were consistent in terms of significance and direction (Anaerostipes, Enterococcus, Faecalibacterium, Lachnospira, Lactobacillus, Parabacteroides, Roseburia) (see Figure 6 and Supplementary Table S9).

**Figure 7:**
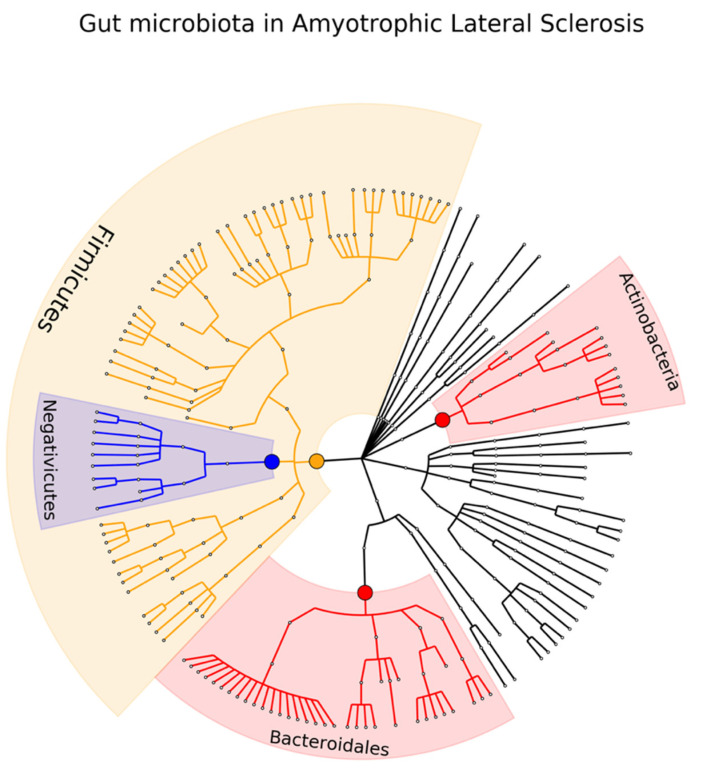
Phylogenetic distributions of the microbiota involved in amyotrophic lateral sclerosis. Orange indicates inconsistent results, blue indicates a decrease in relative abundance, and red indicates an increase in relative abundance. These results are the summary of consistent findings of the included studies [94,95,96,97,98,99].

Six studies on ALS were found, involving a total of 159 ALS patients and 165 healthy controls [94,95,96,97,98,99]. One study did not present statistics but only bar plots to visually represent differences between patients and control so we did not include it in the analysis [100]. Twenty microbiota have been identified in at least one study and only five (20%) were found in two studies. The direction of the association was inconsistent in terms of direction for the phylum Firmicutes and consistent for Class Bacteroidia, Negativicutes, Order Bacteroidales, and Clostridiales. No consistency in association was observed at either the family or the genus level (see Figure 7 and Supplementary Table S10).

### 3.2. Integrating Data across Neurological Diseases

**Figure 8:**
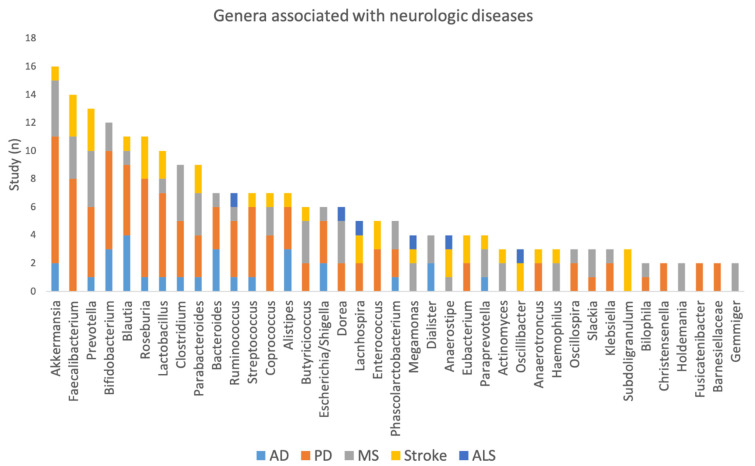
List of the microbiota most frequently found and their distributions according to pathologies.

Figure 8 shows the microbiota that are associated with multiple disorders and the number of studies that found the association of the genus to a disease. Genus Akkermansia is associated with four disorders (AD, PD, MS, and stroke) and Faecalibacterium with three disorders (PD, MS, and stroke). While Prevotella is also found to be associated with four disorders (AD, PD, MS, and stroke), the direction of association is inconsistent across the pathologies and studies.

**Figure 9:**
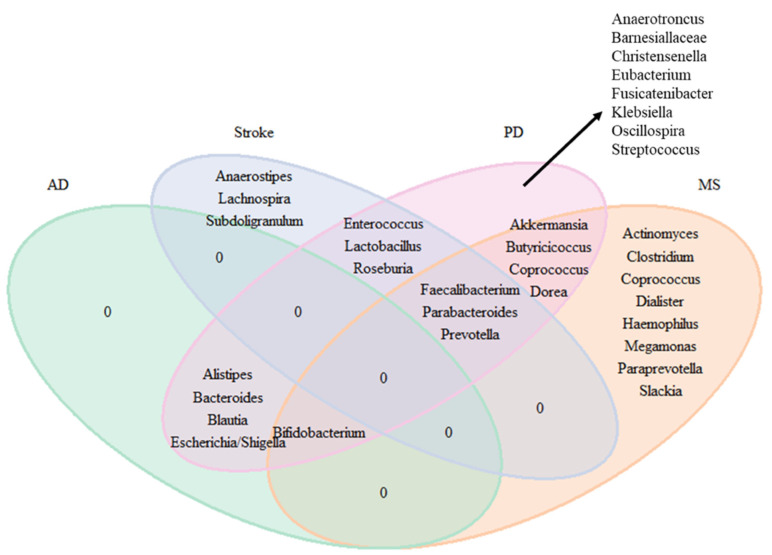
Venn diagram for the genera consistently found in the literature for neurodegenerative and neurological diseases.

In Figure 9, we plotted Venn diagrams summarizing the association to multiple neurological diseases. For the microbiota where inconsistencies were observed across studies, we define consistency if more than 50% of the studies observed association in the same direction. Figure 8 shows that based on the data available to date there is no specific signature for AD or ALS, i.e., no genus involved only in AD or ALS. For PD at the genus level there are eight disease-specific microbiome signatures (Anaerotroncus, Barnesiellaceae, Eubacterium, Fusicatenibacter, Klebsiella, Oscillospira, and Streptococcus). For MS we found eight disease-specific signatures (Actinomyces, Clostridium, Coprococcus, Dialister, Haemophilus, Megamonas, Paraprevotella, and Slackia). Overlapping changes in the microbiome were also seen in PD and MS (Akkermansia, Butyricicoccus, Coprococcus, and Dorea); between PD and AD (Alistipes, Bacteroides, Blautia, and Escherichia/Shigella); between PD and stroke (Enterococcus, Lactobacillus, Roseburia); between PD, MS, and stroke (Faecalibacterium, Parabacteroides, and Prevotella); and between PD, MS, and AD (Bifidobacterium).

**Figure 10:**
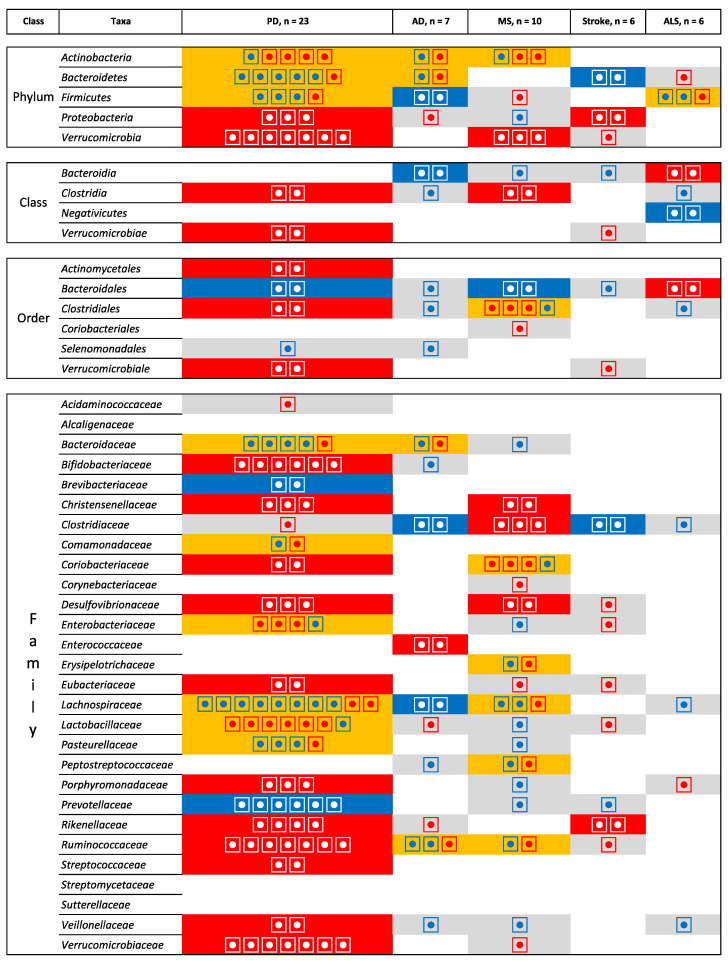
Microbiota that have been identified in at least two studies for the same pathologies at the phylum, class, order and family levels. Grey is used for one study, orange indicates inconsistent results, blue indicates a decrease in relative abundance, and red indicates an increase in relative abundance. The numbers of the circle indicate the number of studies that identify the microbiota and the direction of the inconsistencies.

**Figure 11:**
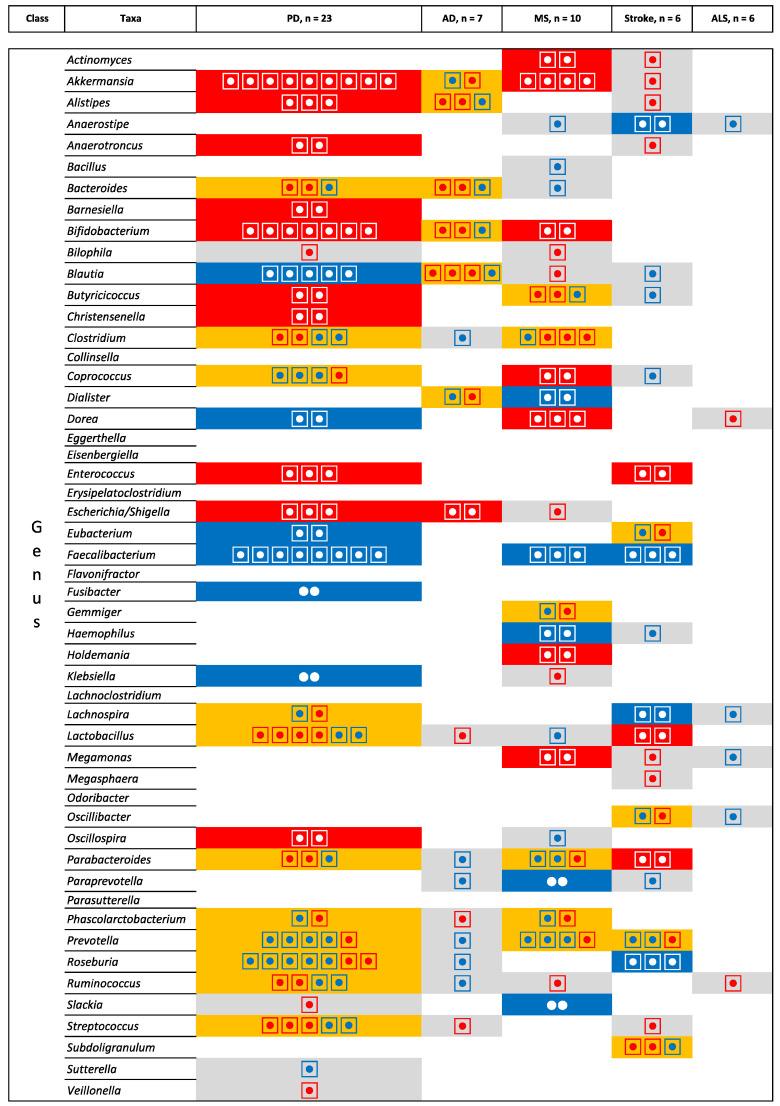
Microbiota that have been identified in at least two studies for the same pathologies at the genus level. Grey is used for one study, orange indicates inconsistent results, blue indicates a decrease in relative abundance, and red indicates an increase in relative abundance. The numbers of the circle indicate the number of studies that identify the microbiota and the direction of the inconsistencies.

However, Figure 8 and Figure 9 do not take into account the direction of the association. Figure 10 and Figure 11 show the direction of the associations. At the genus level, Alistipes, Bacteroides, Bifidobacterium, and Escherichia/Shigella are increased in abundance in AD and PD, while the opposite direction is found for Blautia (relative increase in AD but decrease in PD). When comparing AD to MS there is an increase in the relative abundance of Bifidobacterium. For PD and MS, Akkermansia and Bifidobacterium are found to be consistently increased and Faecalibacterium and Prevotella decreased in abundance. Opposite directions are found for Coprococcus (decrease in PD and increase in MS), Dorea (decrease in PD and increase in MS), and Parabacteroides (increase in PD and decrease in MS). For MS and stroke, there is a decrease in the relative abundance of Faecalibacterium and Prevotella. Finally, when comparing MS to stroke we found a similar direction for Faecalibacterium and Prevotella (increase) and an opposite direction for Parabacteroides (decrease in MS).

The picture emerging is that there is an overlap between neurological disorders for Alistipes, Akkermansia, Blautia, Bifidobacterium, Dorea, Escherichia/Shigella, Faecalibacterium, Parabacteroides, and Prevotella.

## 4. Discussion

The past decade has seen a rapid rise in studies of the role of microbiota in various disorders. In this review, we focus on the consistency of the direction of the relationship found within neurological diseases and between neurologic diseases including PD, AD, ALS, MS, and stroke. The disease with the most consistent microbiome associations is PD. The findings are most reliable as they are based on the largest samples size. Amongst the microbiota associated most frequently, a clear pattern was observed for the genus Akkermansia which showed a consistent increase in nine studies in PD [50,54,55,57,60,61,62,67,69] and four in MS [79,82,83,86]. Akkermansia resides in the mucus layer of the large intestine [101], where they are involved in maintaining intestinal integrity and mucin degradation [102]. Akkermansia uses mucins as a carbon source [103] and can cleave sialic acids [104]. As shown in Figure 2, the increase in the relative abundance of Akkermansia in PD is confirmed at all the taxonomic levels presenting a strong signature of the disease. Such consistency along all the taxonomic levels was not seen for any other microbiota for the other diseases studied. Akkermansia can stimulate dendritic cells to produce TGFβ and interleukin 6 (IL6) and 1 (IL1), activating regulatory T Cells (Tregs) which may be relevant for the pathogenesis of MS as well as for PD [105]. Changes in the gut microbiota have been linked with an increase in inflammation in the central nervous system in various neurological diseases. Our first line of defense is the innate immune system, which consists of physical and chemical barriers, immune cells, and blood proteins (such as cytokines). Toll-like receptors (TLRs) located on the membranes of epithelial and lymphoid cells in the small intestine mediate this differential recognition of commensal and pathogenic bacteria [106]. The question to answer is how to interpret the change in Akkermansia seen in both disorders. MS is primarily a neuroinflammatory disorder and there is increasing interest in the role of neuroinflammation in PD as a therapeutic target [107,108,109]. A late complication of MS and PD is cognitive decline and there is increasing evidence that neuroinflammation is involved in dementia and cognitive decline. In this study, the evidence for the role of Akkermansia in dementia was not consistent. However, a problem interpreting the findings of the AD studies is that the power of these studies has been low and false negative findings may be frequent. Akkermansia have been associated with diabetes and cardiovascular disease, co-morbidity of which are often seen in neurodegenerative disorders. Recently, Akkermansia intervention has been approved by the European Food Safe authority [110]. Last but not least, the abundance of Akkermansia has also been associated with constipation, which is often seen in PD patients and is a major clinical problem [111]. Of note is that constipation is very common in MS patients and a hypothesis to be tested is whether this problem in MS is related to the increased abundance of Akkermansia in the gut.

The overlap with PD and MS is substantial with eight microbiota found in common: Akkermansia, Bifidobacterium, Butyricicoccus, Coprococcus, Dorea, Faecalibacterium, Parabacteroides, and Prevotella. Dorea is abundant in MS but seen to be reduced in PD. The finding of the increased relative abundance of Bifidobacterium across PD and MS studies is far from understood. The genus Bifidobacterium is known to metabolize lactose. The genus Bifidobacterium has previously been implicated in lactose tolerance [112]. Bifidobacterium was the first microbial to colonize humans and is seen as positive for health [113]. For PD, one may speculate that the relatively healthy lifestyle in some patients may partially explain these findings. PD patients have been found to smoke less [114]. The effects of Bifidobacterium and Lactobacillus species (also involved in PD) delivered as probiotics have been previously studied in PD [115]. The results indicated a decrease in negative mood in healthy persons [116]. Psychobiotics are probiotics believed to promote mental wellness through interaction with other commensal gut microbes [117]. In mice, probiotic administration leads to a drop in proinflammatory cytokines and an increase in anti-inflammatory cytokines, resulting in cognitive and emotional enhancement [118]. Furthermore, Dorea is constituent of healthy gut flora and has also been linked to irritable bowel syndrome [119].

The increased abundance of Enterococcus (consistent in all PD samples) and Lactobacillus (abundant in PD patients in most of the included studies) is of interest as these taxa may interfere with Levodopa medication of the patients [120] and thus impact the quality of life [121]. This genus is also implicated in tyrosine metabolism, which is explored as a new intervention in stroke [122]. Interestingly, experimental studies in mice show evidence that the inclusion of Akkermansia and Parabacteroides in the diet of mice confers seizure protection compared to a control diet [123].

Bacteroides was found to be altered in AD and PD. The bacteria are implicated in adaptive immunity, which is mediated by B cells and T cells, two primary lymphocyte populations. Through its polysaccharide molecules, Bacteroides fragilis induces a systemic Th1 response, which is essential for eradicating intracellular infections. On the other hand, segmented filamentous bacteria have been shown to be effective inducers of Th17 cells, and Clostridia have been found to increase the production of colonic Tregs, a crucial mediator of immunological tolerance whose dysfunction can lead to autoimmune diseases. [124]. They can affect the nervous system, contributing to the maturation of naive microglia in the absence of microbiota, which could be explained by the modifications observed in PD and AD.

AD and PD are the two most common neurodegenerative diseases in the elderly. Of note is that each of the microbiota that are consistently associated to AD are also consistently associated to PD. At the genus level, for AD and PD, changes of Alistipes, Bacteroides, Bifidobacterium, and Escherichia/Shigella are found in the same direction (an increase of relative abundance) while the opposite direction is found for Blautia (relative increase in AD but decrease in PD). Alistipes and Bacteroides have been implicated in cholesterol homeostasis. Escherichia coli has been implicated in amyloid (AD) and alpha-synuclein (PD) metabolism as well as gliosis (seen both in AD and PD [104]).

In addition to the pathways discussed above, there are several possible explanations for the mechanisms of action driving the relationship between gut microbiota and the different diseases. A recent review underlines the relationship between inflammation, pain, microbiota, and the different lipids, focusing on the possible involvement of the N-acylethanolamine family and short-chain fatty acids in the gut–brain axis and their role in the central nervous system diseases [125]. The lymphatic system could be the mediator of this communication between the gut microbiota and the brain [126].

In this review, we summarized the current evidence of the modification of gut microbiota in various pathologies. We limited our review to the main neurodegenerative diseases and MS but gut microbiota composition is also modified in other neurological disorders such as Rett syndrome [127] and in neurocritical ill patients [128]. However, their roles are not limited to neurological or neuropsychiatric diseases; they are also involved in hypertension [129,130], cirrhosis [131,132] or primary hepatocellular carcinoma [133], diabetes [134], autoimmune diseases [135], systemic lupus erythematosus [136], systemic immunity in allergic disease [137], Behcet’s disease [138], systemic sclerosis [139], rheumatoid arthritis [140], and could also potentially influence vitamin D production [141]. There are also correlations reported between some gut microbiota and personality in adults [142].

An essential part of the research in this field is the development of interventions to modify the microbiota. The gut microbiome is determined by diet [143,144]. For example, intermittent fasting led to increased gut bacteria richness, enrichment of the Lactobacillaceae, Bacteroidaceae, and Prevotellaceae families, and enhanced antioxidative microbial metabolic pathways [145]. The most promising results of clinical improvement after modification of the gut microbiota have been obtained in patients with epilepsy after a ketogenic diet [146,147,148]. There was an overall decrease in the mean species diversity after treatment and, importantly, a difference in the variation of species between responders and non-responders. Further analysis of species composition before and after treatment showed a significant increase in Bacteroides and a decrease in Firmicutes and Actinobacteria. When comparing responders and non-responders, Clostridiales, Clostridia, Ruminococcaeceae, Lachnospiraceae, Alistipes, and Tikenellacase were significantly increased in non-responders [149]. Interesting clinical results have also been obtained using fecal transplantation (FMT). FMT is the technique of administering feces from healthy donors to potential recipients in attempt to restore a stable microbiota in the stomach [150]. FMT has been shown to be both safe and effective in the treatment of recurrent Clostridium difficile infection [151]. This has prompted research into the impact of FMT on various illnesses brought on or aggravated by gut dysbiosis. Promising results have been found in both animal and human studies [152,153].

Remarkably, FMT reduced gut microbial dysbiosis, decreased fecal SCFAs, alleviated physical impairment, and increased striatal DA and 5-HT content of PD mice. Further, FMT reduced the activation of microglia and astrocytes in the substantia nigra, and reduced expression of TLR4/TNF-α signaling pathway components in gut and brain [152,154].

Another promising domain of research is the modification of gut microbiota composition using probiotics. Probiotics are microorganisms that are administered in the form of a medicine, food, dietary supplement, or infant formula [155]. The majority of a normal probiotic consists of gut bacteria that occur naturally in the human body. They may provide health benefits to the host when administered in sufficient doses and frequency. Probiotic administration is currently under development and validation in various disorders such as PD [156], AD [157], MS [158], and stroke [159].

A lot of research, mainly performed in AD and PD, has also been carried out using transgenic animals (mice) to try to better understand if the changes in abundance in microbiota may be a cause or consequence of pathogenesis as the communication between the microbiome in the gastrointestinal tract and the central nervous system is bi-directional [160]. In AD, different models have been tested: amyloid metabolism (APP/PS1) [39,161,162,163,164,165,166], senescence models (SAMP8) [40,167], and triple mutations (APP, MAPT, PSEN1) [168,169,170]. Of note is that there was no difference between the amyloid models. Studies using animal models also provided new potentially exciting opportunities for the prevention or management of these disorders. In APPswe/PS1dE9 transgenic mice with fecal microbiota transplantation, treatment can improve cognitive deficits and reduce the brain deposition of Aβ. As for translational studies in humans, other approaches have recently been successfully tested in mice to modify the gut microbiota of transgenic animals, such as oral administration of anti-inflammatory Bifidobacterium longum (NK46) from human gut microbiota. NK46 treatment suppressed amyloid-β, β/γ-secretases, and caspase-3 expression and amyloid-β accumulation in the hippocampus of 5XFAD-Tg mice. Suppression of gut dysbiosis by NK46 can mitigate cognitive decline through the regulation of microbiota [171]. Silibinin and silymarin administration tended to decrease the microbiota diversity and exhibited a regulative effect in abundance on several key bacterial species associated with AD development in APP/PS1 mice [172]. In PD, most of the studies have been performed with ASO mice (α-synuclein overexpressing) to stimulate the production of αSyn in the central nervous system [173] and with Methyl-4-phenyl-1,2,3,6-tetrahydropyridine (MPTP) [174]. From a therapeutical perspective, the protective effect of Ceftriaxone has been shown in MPTP intoxicated mouse model. Ceftriaxone had a neuroprotective effect on MPTP-induced PD mice, and its neuroprotective effect could be through regulating inflammation and intestinal microbiota [175]. Other approaches such as curcumin [176], Oligosaccharides [177], chicoric acid [178], and Mucuna pruriens [179] also show promising results.

The main limitation of the current studies are the populations investigated. We have seen that a majority of the studies have been done in China and in the USA but if we analyzed the results by pathologies, we observed that 7 of the 10 studies in MS have been done in the USA and Canada, and 5 out of the 6 studies on stroke in Asia (4 in China, 1 in Japan). It has also been shown that, even within the USA, the gut microbiome composition in PD variated in different states [180]. This high homogeneity generalizes the results for these pathologies, since it has been shown that genetics [181,182], socioeconomic status [183], and diet influence the composition of the microbiota [184,185,186,187].

We observed a major difference in the mean age of the participants in the different pathologies and adjustment for confounding, such as body mass index (BMI), medication, constipation [71,73,77], and/or diet and alcohol consumption [188,189], while it has been demonstrated that those factors influence microbiota composition and abundance [167,190,191,192]. There are also important differences in the types of the different studied diseases. For AD, for example, some studies include patients with very mild dementia and moderate dementia [71]; in one study, the authors compared AD patients with and without amyloidosis [72]. In PD, most of the studies are performed with sporadic PD but also in familial PD, while the genetic factors differ and, therefore, the potential relationship with the brain–gut axis [193]. For studies in MS, there is a mix of patients with relapsing-remitting MS and progressive MS. Last but not least, there are major differences in the severity and in the duration of the disease, two elements that have shown an important role in the alterations of the microbiome in the case of degenerative pathologies. The interpretation is hampered by the low sample sizes of the AD, ALS, and stroke studies leading to false positive and false negative findings.

## 5. Conclusions

Summarizing the evidence for a role of the gut microbiome in and across neurological diseases, we find that the findings are most consistent for PD and MS. We further find that the findings are often not unique to neurological diseases. There is a substantial overlap between PD and MS and AD and PD that needs further investigation about whether this reflects a common etiology of those disorders and shared consequence of neurological pathology. The most remarkable microbiota signature was observed in PD with an important increase in the relative abundance of the Akkermansia at all the different levels of the taxon. Additionally, for MS, findings were often consistent over studies, despite the small series of patients studied. The most similar signature, from the perspectives of microbiome composition, are PD and MS. For future clinical applications, baseline value and standard reporting templates have been developed that should ease and spread the analysis of gut microbiota in daily practice for clinicians [194,195,196]. Despite the limitations of the studies conducted to date, aggregating the data of the current studies strongly suggests that the microbiome is a promising target for preventive and/or restorative treatment. The findings beg for more research aiming to answer the question of whether targeted anti or probiotic interventions may be relevant for the prevention of neurological disorders, or may improve the quality of life of patients.

## Data Availability

Not applicable.

## Supplementary Materials

The following supporting information can be downloaded at: https://www.mdpi.com/article/10.3390/ijms232213665/s1.
