# Supplementary material for: What Are the Key Gut Microbiota Involved in Neurological Diseases? A Systematic Review
Source: Int J Mol Sci. 2022 Nov 8;23(22):13665. doi: 10.3390/ijms232213665 (PMC9696257; doi:10.3390/ijms232213665)
Supplement: Supplementary file 1 [file ijms-23-13665-s001.zip › ijms-1919158-supplementary.pdf]

International Journal of *Molecular Sciences*

Review

What Are the Key Gut Microbiota Involved in Neurological Diseases? A Systematic Review

Bruno Bonnechère [1,2], Najaf Amin [2] and Cornelia van Duijn [2,*]

[1] REVAL Rehabilitation Research Center, Faculty of Rehabilitation Sciences, Hasselt University, 3590 Diepenbeek, Belgium
[2] Nuffield Department of Population Health, University of Oxford, Oxford OX3 7LF, UK
* Correspondence: cornelia.vanduijn@ndph.ox.ac.uk

Supplemenary Materials

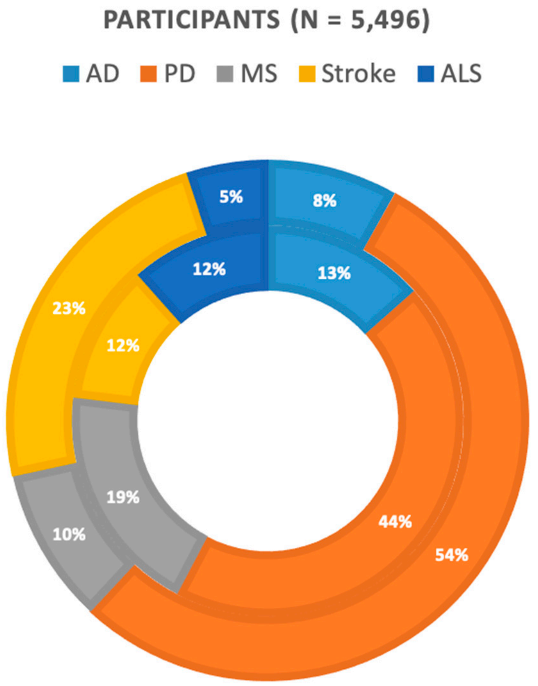

Supplementary Figure S1: Repartition of the patients and the studies.

Citation: Bonnechère, B.; Amin, N.; van Duijn, C. What Are the Key Gut Microbiota Involved in Neurological Diseases? A Systematic Review. *Int. J. Mol. Sci.* **2022**, *23*, 13665. https://doi.org/10.3390/ijms232213665

Academic Editor: Blazej Misiak

Received: 31 August 2022
Accepted: 3 November 2022
Published: 15 November 2022

Publisher's Note: MDPI stays neutral with regard to jurisdictional claims in published maps and institutional affiliations.

Copyright: © 2022 by the authors. Licensee MDPI, Basel, Switzerland. This article is an open access article distributed under the terms and conditions of the Creative Commons Attribution (CC BY) license (https://creativecommons.org/licenses/by/4.0/).

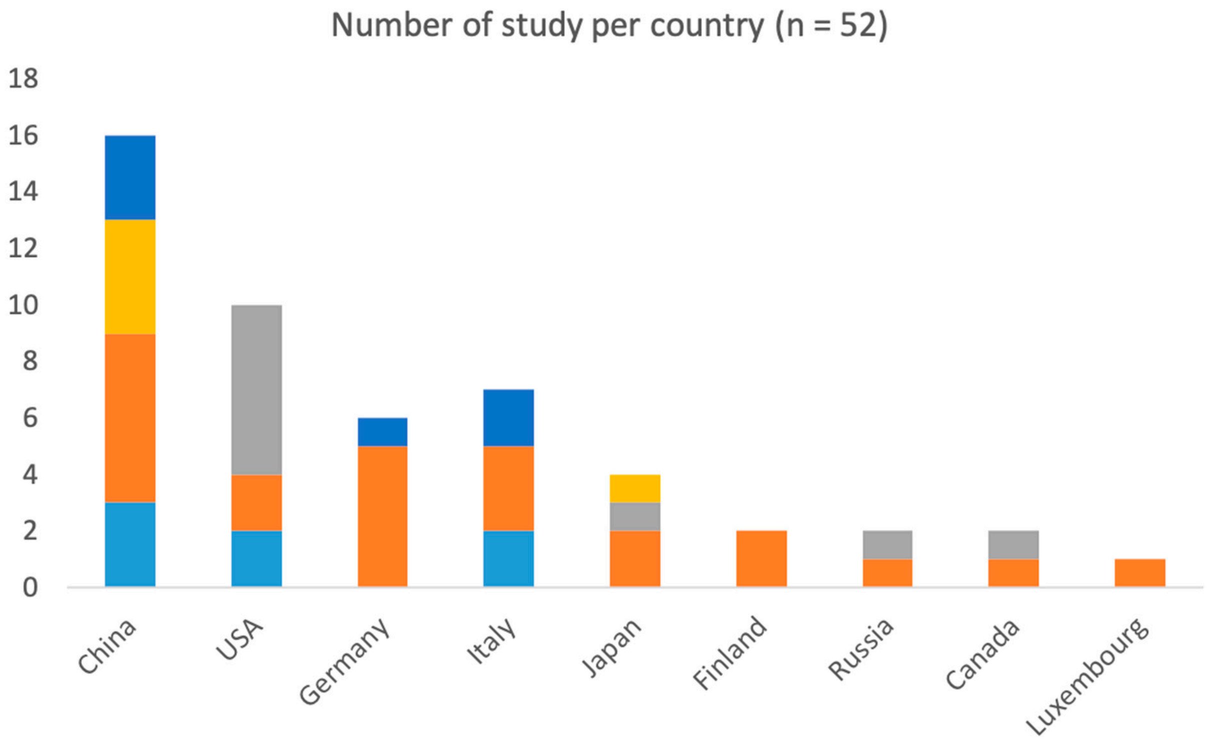

Supplementary Figure S2: Distribution of the included studies according to the countries and studied pathologies.

International Journal of *Molecular Sciences*

MDPI

Supplementary Table S1: Characteristics of the included studies on Parkinson's disease. 1

Study	Country	Participants	Methods
Scheperjans et al. 2015[1]	Finland	72 PD patients (65.3 ± 5 years old, BMI = 26.3 kg/m² [23.3 – 29.3], 35 female)<br>45 controls (64.5 ± 7 years old, BMI = 26.2 kg/m² [23.7 – 28.1], 36 female)	16S rRNA amplicon (V3-V4 regions) sequencing analysis
Hasegawa et al. 2015[2]	Japan	52 PD patients (68.9 ± 7 years old, BMI unknown, 31 female)<br>34 controls (68.4 ± 10 years old, BMI unknown, 15 female)	16S rRNA amplicon (V4 regions) sequencing analysis
Keshavarzian et al. 2015	USA	38 PD patients (61.6 ± 9 years old, BMI = 26.0 ± 3 kg/m², 14 female)<br>34 controls (45.1 ± 14 years old, BMI = 27.6 ± 6 kg/m², 16 female)	16S rRNA amplicon (V4 regions) sequencing analysis
Unger et al. 2016[3]	Germany	34 PD patients (67.7 ± 9 years old, BMI unknown, 10 female)<br>34 controls (64.6 ± 7 years old, BMI unknown, 16 female)	Microbial DNA was extracted using the QIAsymphony DSP Virus/Pathogen Mini-Kit (QIAGEN, Hilden, Germany)
Petrov et al. 2017[4]	Russia	24 PD patients (67 years old [65 – 69.8], BMI = 26.7 kg/m² [25.4 – 28.9], gender not defined)<br>14 controls (63 years old [61.5 – 67.5], BMI = 26.1 kg/m² [23.8 – 28.7], gender not defined)	16S rRNA amplicon (V3-V4 regions) sequencing analysis
Li et al. 2017[5]	China	24 PD patients (73.7 ± 6 years old, BMI = 23.0 ± 4kg/m², 8 female)	Bacterial DNA was extracted using a TIANmp stool DNA kit (Tiangen Biotech Co. Ltd., Beijing). DNA samples were quantified using a Qubit 2.0 Flurometer (Invitrogen, USA)

		14 controls (74.6 ± 6 years old, BMI = 24.0 ± 3 kg/m², 8 female)	
Hill-Burns et al. 2017[6]	USA	197 PD patients (68.4 ± 9 years old, BMI = 26.4 ± 5kg/m², 65 female)<br>130 controls (70.3 ± 9 years old, BMI = 28.3 ± 6 kg/m², 79 female)	16S rRNA amplicon (V4 regions) sequencing analysis
Bedarf et al. 2017[7]	Germany	31 PD patients (64.8 ± 9 years old, BMI unknown, 0 female)<br>28 controls (67.9 ± 9 years old, BMI unknown, 0 female)	Non-invasively obtained stool samples were shotgun sequenced (paired end) using an Illumina Hiseq4000 and further analyzed with the MOCAT2 pipeline
Hopfner et al. 2017[8]	Germany	29 PD patients (69.2 ± 6 years old, BMI unknown, 6 female)<br>29 controls (69.4 ± 7 years old, BMI unknown, 16 female)	16S rRNA amplicon (V1-V2 regions) sequencing analysis
Heintz-Buschart et al. 2018[9]	Luxembourg	76 PD patients (68.0 ± 10 years old, 28.5 ± 4kg/m², 26 female)<br>78 controls (68.4 ± 7 years old, 26.6 ± 4kg/m², 32 female)	16S and 18S rRNA amplicon (V4 regions) sequencing analysis
Qian et al. 2018[10]	China	45 PD patients (68.1 ± 8 years old, BMI = 22.8 ± 2kg, 23 female)<br>45 controls (67.9 ± 9 years old, BMI = 23.4 ± 2kg, 22 female)	16S rRNA amplicon (V3-V4 regions) sequencing analysis
Lin et al. 2018[11]	China	74 PD patients (60.5 ± 11 years old, BMI = 24.3 ± 2kg, 26 female)<br>44 controls (63.2 ± 6 years old, BMI = 24.4 ± 2kg, 22 female)	16S rRNA amplicon (V4 regions) sequencing analysis
Barichella et al. 2018[12]	Italy	193 PD patients (67.6 ± 9.7 years old, BMI = 25.2 ± 4.6, 78 female)<br>113 controls (65.9 ± 9.9 years old, BMI = 25.3 ± 4.6, 66 female)	16S rRNA amplicon (V4 regions) sequencing analysis
Li et al. 2019[13]	China	51 PD patients (62.4 ± 8 years old, BMI = 24.9 ± 4kg, 19 female)	16S rRNA amplicon sequencing analysis

		48 controls (62.2 ± 9 years old, BMI = 24.5 ± 3kg, 29 female)	
Li et al. 2019[14]	China	10 PD patients (79.5 ± 8 years old, BMI = 23.4 ± 3kg, 3 female) 48 controls (76.5 ± 7 years old, BMI = 23.5 ± 4kg, 5 female)	16S rRNA amplicon (V4 regions) sequencing analysis
Aho et al. 2019[15]	Finland	64 PD patients (64.4 ± 7 years old, BMI = 26.2 ± 2kg, 32 female) 64 controls (76.5 ± 7 years old, BMI = 26.5 ± 2kg, 31 female)	16S rRNA amplicon (V4 regions) sequencing analysis
Weis et al. 2019[16]	Germany	34 PD patients (65.2 ± 7 years old, BMI unknown, 11 female) 25 controls (63.9 ± 8 years old, BMI unknown, 14 female)	16S rRNA amplicon (V4-V5 regions) sequencing analysis
Pietrucci et al. 2019[17]	Italy	80 PD patients (64.4 ± 7 years old, BMI = 26.2 ± 2kg, 32 female) 72 controls (76.5 ± 7 years old, BMI = 26.5 ± 2kg, 31 female)	16S rRNA amplicon (V4 regions) sequencing analysis
Ren et al. 2020[18]	China	27 PD patients (62.3 ± 9 years old, BMI = 22.6 ± 2kg, 8 female) 13 controls (63 ± 9 years old, BMI = 22.7 ± 2kg, 10 female)	16S rRNA amplicon (V4 regions) sequencing analysis
Vascellari et al. 2020[19]	Italy	64 PD patients (71.4 ± 11 years old, BMI = 26.1 ± 4kg, 20 female) 51 controls (51.7 ± 12 years old, BMI = 23.7 ± 3kg, 20 female)	16S rRNA amplicon (V4 regions) sequencing analysis
Cosma-Grigorov et al. 2020[20]	Germany	71 PD patients (65.3 ± 10 years old, BMI = 26.1 ± 4kg, 37 female) 30 controls (64.3 ± 9 years old, BMI = 26.0 ± 3kg, 16 female)	16S rRNA amplicon (V3-V4 regions) sequencing analysis
Nishiwaki et al. 2020[21]	Japan	223 PD patients (68.3 ± 10 years old, BMI = 22.9 ± 3kg, 128 female)	16S rRNA amplicon (V3-V4 regions) sequencing analysis

		117 controls (68.2 ± 9 years old, BMI = 21.7 ± 3kg, 71 female)	
Cirstea et al. 2020[22]	Canada	197 PD patients (66 [59 - 71] years old, BMI = 26.1 [22.9 – 29.1] kg, 75 female)<br>103 controls (66 [58 - 71] years old, BMI = 26.2 [23.1 – 29.5] kg, 52 female)	16S rRNA amplicon (V3-V4 regions) sequencing analysis

Supplementary Table S2: Characteristics of the included studies on Alzheimer's disease.

Study	Country	Participants	Methods
Vogt et al. 2017[23]	USA	25 AD patients (71±7 years old, 26.1 [24.3 – 33.2] kg/m^2,17 female) and 25 control (69±7 years old, 26.0 [22.9 – 29.1] kg/m^2, 18 female).	16S rRNA amplicon (V3-V4 regions) sequencing analysis
Cattaneo et al. 2017[24]	Italy	40 AD patients with brain amyloidosis (71±7 years old, 24.5±3.5kg/m^2,20 female), 33 AD patients without brain amyloidosis (70±7 years old, 25.6±3.7kg/m^2, 18 female) and 10 control (68±8 years old, 24.3±2.9kg/m^2, 6 female).	16S rRNA amplicon (V3-V4 regions) sequencing analysis
Zhuang et al. 2018[25]	China	43 AD patients (70±9 years old, unknown BMI,20 female) and 43 control (71±7 years old, unknown BMI, 20 female)	16S rRNA amplicon (V3-V4 regions) sequencing analysis
Haran et al. 2019[26]	USA	24 AD patients (85±8 years old, unknown BMI, 20 female) and 51 control (83±10 years old, unknown BMI, 43 female)	16S rRNA amplicon (V3-V4 regions) sequencing analysis

Lopizzo et al. 2019[27]	Italy	30 AD patients and 25 controls	Bacterial DNA was isolated from stool and 16S genes have been sequenced onto MiSeq platform
Liu et al. 2019[28]	China	33 AD patients (75±11 years old, 22.0±1.3kg/m^2, 14 female) and 32 control (77±9 years old, 22.2±2.3kg/m^2, 16 female)	16S rRNA amplicon (V3-V4 regions) sequencing analysis Genomic DNA
Li et al. 2019[29]	China	30 AD patients (66±5 years old, 23.0±3.5kg/m^2, 15 female) and 30 controls (64±5 years old, 24.0±2.9kg/m^2, 17 female)	16S rRNA amplicon (V3-V4 regions) sequencing analysis

Supplementary Table S3: Characteristics of the included studies on Multiple Sclerosis.

Study	Country	Participants	Methods
Miyake et al. 2015[30]	Japan	20 MS patients (36.0 ± 7 years old, BMI unknown, 14 female) 40 control (27. ± 2 9 years old, BMI unknown, 18 female)	16S rRNA amplicon (V1-V2 regions) sequencing analysis
Cantarel et al. 2015[31]	USA	15 MS patients (42 [30 – 48] years old, BMI unknown, 14 female) 8 control (38 [29 – 51] years old, BMI unknown, 18 female)	16S rRNA amplicon (V3-V4 regions) sequencing analysis
Chen et al. 2016[32]	USA	31 MS patients (42.9 ± 11 years old, 28.0 ± 6 kg/m^2, 21 female) 36 control (40.3 ± 7 years old, 27.4 ± 5 kg/m^2, 14 female)	16S rRNA amplicon (V3-V5 regions) sequencing analysis
Tremlet et al. 2016[33,34]	USA	18 pediatric MS patients (12.5 ± 4 years old, 22.2 ± 6 kg/m^2, 10 female) 17 control (12.8 ± 7 years old, 27.4 ± 5 kg/m^2, 9 female)	16S rRNA amplicon (V4 regions) sequencing analysis
Jangi et al. 2016[35]	USA	60 MS patients (49.7 ± 8 years old, 27.2 ± 6 kg/m^2, 41 female)	16S rRNA amplicon (V3-V5 regions) sequencing analysis

		43 control (42.2 ± 10 years old, 26.4 ± 6 kg/m^2, 37 female)	
Cekanaiciute et al. 2017[36]	USA	71 MS patients (52.5 ± 8 years old, 25.3 ± 6 kg/m^2, 43 female) 71 control (54.3 ± 9 years old, 24.9 ± 5 kg/m^2, 29 female)	16S rRNA amplicon (V4 regions) sequencing analysis
Forbes et al. 2018[37]	Canada	19 MS patients (47.3 years old, BMI unknow, 14 female) 23 control (32.4 years old, BMI unknow, 12 female)	16S rRNA amplicon (V4 regions) sequencing analysis
Kozhieva et al. 2019[38]	Russia	15 MS patients (23 [20 – 73] years old, 24 [17 – 30] kg/m^2, 7 female) 15 control (45 [25 – 56] years old, 22 [20 – 27] kg/m^2, 7 female)	16S rRNA amplicon (V3–V4 regions) sequencing analysis
Ventura et al. 2019[39]	USA	45 MS patients (37.1 ± 13 years old, BMI unknow, 34 female) 44 control (31.8 ± 9 years old, BMI unknow, 28 female)	16S rRNA amplicon (V4 regions) sequencing analysis
Oezguen et al. 2019[40]	Turkey	13 MS patients (39.1 ± 12 years old, BMI unknow, 8 female) 14 control (37.8 ± 9 years old, BMI unknow, 10 female)	16S rRNA amplicon (V3-V4 regions) sequencing analysis

Supplementary Table S4: Characteristics of the included studies on Stroke. 7

Study	Country	Participants	Methods
Yin et al. 2015[41]	China	141 patients with cerebral ischemic stroke (61.0 ± 19 years old, 23.6 ± 4 kg/m², 9 female) 94 control (56 ± 11 years old, 25.1 ± 4 kg/m², 12 female).	16S rRNA amplicon (V4 regions) sequencing analysis
Yamashiro et al. 2017[42]	Japan	41 patients with acute ischemic stroke (65.4 ± 14 years old, 23.5 kg/m² [21.5 – 26.0], 10 female) 40 control (67.4 ± 9 years old, 23.4 kg/m² [21.6 – 26.0], 16 female).	16Sand 23S rRNA amplicon sequencing analysis
Xia et al. 2019[43]	China	104 patients with large-artery atherosclerotic ischemic stroke (59.4 ± 13 years old, BMI unknown, 26 female) 90 control (56.6 ± 8 years old, BMI unknown, 17 female).	16S rRNA amplicon (V4 regions) sequencing analysis
Li et al. 2019[44]	China	30 patients with cerebral ischemic stroke (60.5 ± 11 years old, BMI unknown, 9 female) 30 control (64.2 ± 13 years old, BMI unknown, 12 female).	16S rRNA amplicon (V4 regions) sequencing analysis
Li et al. 2020[45]	China	79 patients with cerebral infarction (66.1 ± 12 years old, BMI 24.2± 2 kg/m², 29 female) 98 control (64.0 ± 10 years old, BMI 23.8 ± 2 kg/m², 41 female).	16S rRNA amplicon (V4 regions) sequencing analysis
Haak et al. 2020[46]	The Netherland	349 patients with stroke (72 [62 - 80] years old, BMI unknown, 155 female) 98 control (64.0 ± 10 years old, BMI unknown, 21 female).	16S rRNA amplico<br>n (V3-V4 regions) sequencing analysis

Supplementary Table S5: Characteristics of the included studies on Amyotrophic Lateral Sclerosis.

Study	Country	Participants	Methods
Fang et al. 2016[47]	China	6 ALS patients (56.8 ± 11 years old, BMI unknown, 1 female)<br>5 control (49.6 ± 5 years old, BMI unknown, 3 female)	16S rRNA amplicon (V3-V4 regions) sequencing analysis
Brenner et al. 2018[48]	Germany	25 ALS patients (57.6 ± 11 years old, 25.6 ± 5 kg/m^2, 13 female)<br>32 control (56.0 ± 12 years old, 25.7 ± 4 kg/m^2, 16 female)	16S rRNA amplicon (V3-V6 regions) sequencing analysis
Mazzini et al. 2018[49]	Italy	50 ALS patients (57.6 ± 11 years old, 25.6 ± 5 kg/m^2, 13 female)<br>50 control (56.0 ± 12 years old, 25.7 ± 4 kg/m^2, 16 female)	16S rRNA amplicon (V3-V4 regions) sequencing analysis
Zhai et al. 2019[50]	China	8 ALS patients (57.4 ± 13 years old, 20.3 ± 2 kg/m^2, 4 female)<br>8 control (50.8 ± 5 years old, 24.9 ± 1 kg/m^2, 4 female)	16S rRNA amplicon (V4-V5 regions) sequencing analysis
Zeng et al. 2020[51]	China	20 ALS patients (53.9 ± 10 years old, 20.3 ± 2 kg/m^2, 8 female)<br>20 control (50.6 ± 13 years old, 24.9 ± 1 kg/m^2, 8 female)	16S rRNA amplicon (V4 region) sequencing analysis
Di Gioia et al. 2020[52]	Italy	50 ALS patients (60.2 ± 11 years old, 23.3 ± 4 kg/m^2, 22 female)<br>50 control (53.6 ± 15 years old, 24.1 ± 4 kg/m^2, 22 female)	16S rRNA amplicon (V3-V4 regions) sequencing analysis

International Journal of *Molecular Sciences*

MDPI

Supplementary Table S6: Individual results of the included studies on Parkinson's disease.

Class	Taxa	Study: Scheperjans et al. 2015	Hasegawa et al. 2015	Keshavarzian et al. 2015	Unger et al. 2016	Petrov et al. 2017	Li et al. 2017	Hill-Burns et al. 2017	Bedarf et al. 2017	Hopfner et al. 2017	Heintz-Buschart et al. 2018	Qian et al. 2018	Lin et al. 2018	Barichella et al. 2018	Li et al. 2019a	Li et al. 2019b	Aho et al. 2019	Weis et al. 2019	Pietrucci et al. 2019
	N patients	72	52	38	34	24	24	197	31	29	76	45	74	193	51	10	64	34	80
	N controls	72	36	34	34	14	14	130	28	29	78	45	44	113	48	10	64	25	72
Phylum	*Verrucomicrobia*																		
	Bacteroides																		
	Firmicutes																		
	Actinobacteria																		
	Euryarchaeota																		
	Proteobacteria																		
	Tenericutes																		
Class	*Clostridia*																		
	Verrucomicrobia																		
	Bacilli																		
	Negativicutes																		
Order	*Actinomycetales*																		
	Clostridiales																		
	Verrucomicrobiale																		
	Bacteroidales																		
	Enterobacteriales																		
	Lactobacillales																		
	Selenomonadales																		
Family	*Bacteroidaceae*																		
	Bifidobacteriaceae																		
	Christensenellaceae																		
	Porphyromonadaceae																		
	Prevotellaceae																		
	Rikenellaceae																		
	Ruminococcaceae																		
	Verrucomicrobiaceae																		
	Enterobacteriaceae																		
	Lachnospiraceae																		
	Lactobacillaceae																		
	Pasteurellaceae																		
	Acidaominococcaceae																		
	Actinomycetaceae																		
	Aerococcaceae																		
	Bradyrhizobiaceae																		
	Brevibacteriaceae																		
	Brucellaceae																		
	Clostridiaceae																		
	Comamonadaceae																		
	Desulfovibrionaceae																		
	Enterococcaceae																		
	Eubacteriaceae																		
	Gemellaceae																		
	Halmonadaceae																		
	Hyphomonadaceae																		
	Idiomarinaceae																		
	Intrasporangiacea																		
	Leuconostocaceae																		
	Methanobacteriacea																		
	Methylobactericeae																		
	Micrococcaceae																		
	Puniceicoccaceae																		
	Sphingomonadaceae																		
	Streptococcaceae																		
	Veillonellaceae																		
	Xanthomonadaceae																		
Genus	*Akkermansia*																		
	Alistipes																		
	Anaerotruncus																		
	Bifidobacterium																		
	Blautia																		
	Dorea																		
	Enterococcus																		
	Escherichia/Shigella																		
	Eubacterium																		
	Faecalibacterium																		
	Oscillospira																		
	Parabacteroides																		
	Bacteroides																		
	Clostridium																		
	Coprococcus																		
	Lactobacillus																		
	Phascolarctobacterium																		
	Prevotella																		
	Roseburia																		
	Ruminococcus																		
	Streptococcus																		
	Aquabacterium																		
	Barnesiellaceae																		
	Butyricicoccus																		
	Capnocytophaga																		
	Catabacter																		
	Christensenella																		
	Fusicatenibacter																		
	Holdemania																		
	Hydrogenoanaerobacterium																		
	Klebsiella																		
	Lachnospira																		
	Methanobrevibacter																		
	Peptoniphilus																		
	Proteus																		
	Pseudomonas																		
	Sphingonomas																		
	Staphylococcus																		

Supplementary Table S7: Individual results of the included studies on Alzheimer's disease.

Class	Taxa	Study<br>Vogt *et al.* 2017	Cattaneo *et al.* 2017	Zhuang *et al.* 2018	Haran *et al.* 2019	Lopizzo *et al.* 2019	Liu *et al.* 2019	Li *et al.* 2019
	N patients	**25**	**73**	**43**	**24**	**30**	**33**	**30**
	N controls	**25**	**10**	**43**	**51**	**25**	**32**	**30**
Phylum	*Firmicutes*							
	Actinobacteria							
	Bacteroidetes							
	Proteobacteria							
Class	*Bacteroidia*							
	Actinobacteria							
	Bacilli							
	Negativicutes							
	Clostridia							
	Gammaproteobacteria							
Order	*Bacteroidales*							
	Clostridiales							
	Enterobacteriales							
	Lactobacillales							
	Selenomonadales							
Family	*Clostridiaceae*							
	Enterococcaceae							
	Lachnospiraceae							
	Bacteroidaceae							
	Ruminococcaceae							
	Bifidobacteriaceae							
	Gemellaceae							
	Lactobacillaceae							
	Mogibacteriaceae							
	Peptostreptococcaceae							
	Rikenellaceae							
	Turicibacteraceae							
	Veillonellaceae							
Genus	*Escherichia/Shigella*							
	Akkermansia							
	Alistipes							
	Bacteroides							
	Bifidobacterium							
	Blautia							
	Dialister							
	Adlercreutzia							
	Bacteroides Fragilis							
	Barnesiella							
	Bilophia							
	cc115							
	Citrobacter							
	Clostridium							
	Dorea							
	Eubacterium							
	Gemella							
	Lachnoclostridium							
	Lactobacillus							
	Odoribacter							
	Oxalobacter							
	Parabacteroides							
	Paraprevotella							
	Parasutterella							
	Phascolarctobacterium							
	Prevotella							
	Roseburia							
	Ruminococcus							
	SMB53							
	Streptococcus							
	Subdoligranum							
	Sutterella							
	Turicibacter							

Supplementary Table S8: Individual results of the included studies on Multiple Sclerosis.

Class	Taxa	Study<br>Miyake et al. 2015	Cantarel et al. 2015	Chen et al. 2016	Tremlet et al. 2016 (Pediatric MS)	Jangi et al. 2016	Cekanaiciute et al. 2017	Forbes et al. 2018	Kozhieva et al. 2019	Ventura et al. 2019	Oezguen et al. 2019
	N patients	20	15	31	18	60	71	19	15	45	13
	N controls	40	8	36	17	43	71	23	15	44	14
Phylum	*Verrucomicrobia*										
	Actinobacteria										
	Euryarchaeota										
	Firmicutes										
	Proteobacteria										
Class	*Clostridia*										
	Actinobacteria										
	Bacteroidia										
Order	*Bacteroidales*										
	Clostridiales										
	Coriobacteriales										
Family	*Clostridiaceae*										
	Christensenellaceae										
	Desulfovibrionaceae										
	Coriobacteriaceae										
	Erysipelotrichaceae										
	Lachnospiraceae										
	Peptostreptococcaceae										
	Pseudomonadaceae										
	Ruminococcaceae										
	Acidaminococcaceae										
	Actinomycetaceae										
	Bacteroidaceae										
	Bdellovibrionaceae										
	Caulobactereceae										
	Corynebacteriaceae										
	Enterobacteriaceae										
	Eubacteriaceae										
	Flavobacteriaceae										
	Lactobacillaceae										
	Oxalobacteraceae										
	Pasteurellaceae										
	Porphyromonadaceae										
	Prevotellaceae										
	Sphingomonadaceae										
	Veillonellaceae										
	Verrucomicrobiaceae										
Genus	*Actinomyces*										
	Akkermansia										
	Bifidobacterium										
	Coprococcus										
	Dialister										
	Dorea										
	Faecalbacterium										
	Haemophilus										
	Megasphaera										
	Paraprevotella										
	Slackia										
	Butyricicoccus										
	Clostridium										
	Gemmiger										
	Parabacteroides										
	Phascolarctobacterium										
	Prevotella										
	Acidaminococcus										
	Acinetobacter										
	Adlercreutzia										
	Anaerofustis										
	Anaerostipes										
	Aquamonas										
	Bacillus										
	Bacteroides										
	Bilophilia										
	Blautia										
	Bulleidia										
	Butyricimonas										
	Catenibacterium										
	Cloacibacillus										
	Collinsella										
	Coprobacillus										
	Coriobacteriaceae										
	Corynebacterium										
	Escherichia/Shigella										
	Faecalicoccus										
	Flavobacterium										
	Fusivatenibacter										
	Holdemania										
	Klebsiella										
	Lachnospira										
	Lactobacillus										
	Megamonas										
	Methanobrevibacter										
	Mogibacterium										
	Molliuts										
	Moraxellaceae										
	Mycoplana										
	Oscillospira										
	Pedobacter										
	Pseudomonas										
	Ruminococcus										
	Sarcina										
	Serratia										
	Sporobacter										
	Streptococcus										
	Subdoligranulum										
	Succinivibro										
	Turicibacter										
	Variabaculum										

Supplementary Table S9: Individual results of the included studies on Stroke.

Class	Taxa	Study: Yin et al. 2015	Study: Yamashiro et al. 2017	Study: Xia et al. 2019	Study: Li et al. 2019
	N patients	141	41	104	30
	N controls	94	40	90	30
Phylum	*Bacteroidetes*	[blue]			
	Proteobacteria	[red]			
	Verrucomicrobia				[red]
	Parcubacteria				[red]
Class	*Bacteroidia*	[blue]			
	Verrucomicrobia				[red]
	Parcubacteria				[red]
	Synergistia	[red]			
Order	*Bacteroidales*	[blue]			
	Verrucomicrobiale				[red]
	Synergistales	[red]			
Family	*Clostridiaceae*			[blue]	[blue]
	Rikenellaceae	[red]		[red]	
	Bradyrhizobiaceae			[blue]	
	Caulobacteraceae			[blue]	
	Desulfovibrionaceae	[red]			
	Enterobacteriaceae			[red]	
	Eubacteriaceae	[red]			
	Lactobacillaceae	[red]			
	Oscillospiraceae			[red]	
	Porphyromadaceae	[red]			
	Prevotellaceae	[blue]			
	Ruminococcaceae			[red]	
	Shewanellaceae	[blue]			
	Synergistaceae	[red]			
Genus	*Faecalbacterium*	[blue]		[blue]	
	Parabacteroides	[red]		[red]	
	Prevotella	[blue]		[blue]	
	Akkermansia				[red]
	Alistipes	[red]			
	Anaerosporobacter	[blue]			
	Anaerostipes				[blue]
	Atopobium		[red]		
	Bilophila			[red]	
	Butyricimonas			[red]	
	Coccoides		[blue]		
	Coprococcus			[blue]	
	Deltaproteobacteria	[red]			
	Desulfovibrio	[red]			
	Desulfovibrionales	[red]			
	Enterobacter	[red]			
	Enterococcus		[red]		
	Eubacterium	[red]			
	Flavobacteria				[red]
	Fragilis		[blue]		
	Haemophilus			[blue]	
	Knoellia			[blue]	
	Lachnospira			[blue]	
	Lactobacillus	[red]			
	Megasphaera	[red]			
	Odoribacter				[red]
	Oscillibacter	[red]			
	Paraprevotella	[blue]			
	Parcubacteria				[red]
	Roseburia			[blue]	
	Ruminiclostridium				[blue]
	Ruminococcaceae				[red]
	Shewanella	[blue]			
	Subdoligranulum	[red]			
	Synergistetes	[red]			
	Victivallis				[red]

Supplementary Table S10: Individual results of the included studies on Amyotrophic Lateral Sclerosis.

Class	Taxa	Study			
		Fang et al. 2016	Brenner et al. 2018	Mazzini et al. 2018	Zhai et al. 2019
	N patients	**6**	**25**	**50**	**8**
	N controls	**5**	**32**	**50**	**8**
Phylum	*Firmicutes*				
	Bacteroides				
	Euryarchaeota				
Class	*Bacilli*				
	Bacteroidia				
	Clostridia				
	Negativicutes				
Order	*Bacteroidales*				
	Clostridiales				
	Enterobacteriales				
Family	*Enterobacteriaceae*				
	Lachnospiraceae				
	XIII				
Genus	*Anaerostipes*				
	Dorea				
	Lachnospira				
	Methanobrevibacter				
	Oscillibacter				
	Ruminococcus				

References

1. Scheperjans, F.; Aho, V.; Pereira, P.A.B.; Koskinen, K.; Paulin, L.; Pekkonen, E.; Haapaniemi, E.; Kaakkola, S.; Eerola-Rautio, J.; Pohja, M.; et al. Gut Microbiota Are Related to Parkinson's Disease and Clinical Phenotype. *Mov. Disord.* **2015**, *30*, 350–358, doi:10.1002/mds.26069.
2. Hasegawa, S.; Goto, S.; Tsuji, H.; Okuno, T.; Asahara, T.; Nomoto, K.; Shibata, A.; Fujisawa, Y.; Minato, T.; Okamoto, A.; et al. Intestinal Dysbiosis and Lowered Serum Lipopolysaccharide-Binding Protein in Parkinson's Disease. *PLOS ONE* **2015**, *10*, e0142164, doi:10.1371/journal.pone.0142164.
3. Unger, M.M.; Spiegel, J.; Dillmann, K.-U.; Grundmann, D.; Philippeit, H.; Bürmann, J.; Faßbender, K.; Schwiertz, A.; Schäfer, K.-H. Short Chain Fatty Acids and Gut Microbiota Differ between Patients with Parkinson's Disease and Age-Matched Controls. *Parkinsonism Relat. Disord.* **2016**, *32*, 66–72, doi:10.1016/j.parkreldis.2016.08.019.
4. Petrov, V.A.; Saltykova, I.V.; Zhukova, I.A.; Alifirova, V.M.; Zhukova, N.G.; Dorofeeva, Y.B.; Tyakht, A.V.; Kovarsky, B.A.; Alekseev, D.G.; Kostryukova, E.S.; et al. Analysis of Gut Microbiota in Patients with Parkinson's Disease. *Bull. Exp. Biol. Med.* **2017**, *162*, 734–737, doi:10.1007/s10517-017-3700-7.
5. Li, W.; Wu, X.; Hu, X.; Wang, T.; Liang, S.; Duan, Y.; Jin, F.; Qin, B. Structural Changes of Gut Microbiota in Parkinson's Disease and Its Correlation with Clinical Features. *Sci China Life Sci* **2017**, *60*, 1223–1233, doi:10.1007/s11427-016-9001-4.
6. Hill-Burns, E.M.; Debelius, J.W.; Morton, J.T.; Wissemann, W.T.; Lewis, M.R.; Wallen, Z.D.; Peddada, S.D.; Factor, S.A.; Molho, E.; Zabetian, C.P.; et al. Parkinson's Disease and Parkinson's Disease Medications Have Distinct Signatures of the Gut Microbiome. *Mov. Disord.* **2017**, *32*, 739–749, doi:10.1002/mds.26942.
7. Bedarf, J.R.; Hildebrand, F.; Coelho, L.P.; Sunagawa, S.; Bahram, M.; Goeser, F.; Bork, P.; Wüllner, U. Functional Implications of Microbial and Viral Gut Metagenome Changes in Early Stage L-DOPA-Naïve Parkinson's Disease Patients. *Genome Med* **2017**, *9*, 39, doi:10.1186/s13073-017-0428-y.
8. Hopfner, F.; Künstner, A.; Müller, S.H.; Künzel, S.; Zeuner, K.E.; Margraf, N.G.; Deuschl, G.; Baines, J.F.; Kuhlenbäumer, G. Gut Microbiota in Parkinson Disease in a Northern German Cohort. *Brain Res.* **2017**, *1667*, 41–45, doi:10.1016/j.brainres.2017.04.019.
9. Heintz-Buschart, A.; Pandey, U.; Wicke, T.; Sixel-Döring, F.; Janzen, A.; Sittig-Wiegand, E.; Trenkwalder, C.; Oertel, W.H.; Mollenhauer, B.; Wilmes, P. The Nasal and Gut Microbiome in Parkinson's Disease and Idiopathic Rapid Eye Movement Sleep Behavior Disorder. *Mov. Disord.* **2018**, *33*, 88–98, doi:10.1002/mds.27105.
10. Qian, Y.; Yang, X.; Xu, S.; Wu, C.; Song, Y.; Qin, N.; Chen, S.-D.; Xiao, Q. Alteration of the Fecal Microbiota in Chinese Patients with Parkinson's Disease. *Brain Behav. Immun.* **2018**, *70*, 194–202, doi:10.1016/j.bbi.2018.02.016.
11. Lin, A.; Zheng, W.; He, Y.; Tang, W.; Wei, X.; He, R.; Huang, W.; Su, Y.; Huang, Y.; Zhou, H.; et al. Gut Microbiota in Patients with Parkinson's Disease in Southern China. *Parkinsonism Relat. Disord.* **2018**, *53*, 82–88, doi:10.1016/j.parkreldis.2018.05.007.
12. Barichella, M.; Severgnini, M.; Cilia, R.; Cassani, E.; Bolliri, C.; Caronni, S.; Ferri, V.; Cancello, R.; Ceccarani, C.; Faierman, S.; et al. Unraveling Gut Microbiota in Parkinson's Disease and Atypical Parkinsonism. *Movement Disorders* **2019**, *34*, 396–405, doi:10.1002/mds.27581.
13. Li, C.; Cui, L.; Yang, Y.; Miao, J.; Zhao, X.; Zhang, J.; Cui, G.; Zhang, Y. Gut Microbiota Differs Between Parkinson's Disease Patients and Healthy Controls in Northeast China. *Front. Mol. Neurosci.* **2019**, *12*, doi:10.3389/fnmol.2019.00171.
14. Li, F.; Wang, P.; Chen, Z.; Sui, X.; Xie, X.; Zhang, J. Alteration of the Fecal Microbiota in North-Eastern Han Chinese Population with Sporadic Parkinson's Disease. *Neuroscience Letters* **2019**, *707*, 134297, doi:10.1016/j.neulet.2019.134297.
15. Aho, V.T.E.; Pereira, P.A.B.; Voutilainen, S.; Paulin, L.; Pekkonen, E.; Auvinen, P.; Scheperjans, F. Gut Microbiota in Parkinson's Disease: Temporal Stability and Relations to Disease Progression. *EBioMedicine* **2019**, *44*, 691–707, doi:10.1016/j.ebiom.2019.05.064.
16. Weis, S.; Schwiertz, A.; Unger, M.M.; Becker, A.; Faßbender, K.; Ratering, S.; Kohl, M.; Schnell, S.; Schäfer, K.-H.; Egert, M. Effect of Parkinson's Disease and Related Medications on the Composition of the Fecal Bacterial Microbiota. *npj Parkinson's Disease* **2019**, *5*, 1–9, doi:10.1038/s41531-019-0100-x.
17. Pietrucci, D.; Cerroni, R.; Unida, V.; Farcomeni, A.; Pierantozzi, M.; Mercuri, N.B.; Biocca, S.; Stefani, A.; Desideri, A. Dysbiosis of Gut Microbiota in a Selected Population of Parkinson's Patients. *Parkinsonism Relat. Disord.* **2019**, *65*, 124–130, doi:10.1016/j.parkreldis.2019.06.003.

18. Ren, T.; Gao, Y.; Qiu, Y.; Jiang, S.; Zhang, Q.; Zhang, J.; Wang, L.; Zhang, Y.; Wang, L.; Nie, K. Gut Microbiota Altered in Mild Cognitive Impairment Compared With Normal Cognition in Sporadic Parkinson's Disease. *Front Neurol* **2020**, *11*, 137, doi:10.3389/fneur.2020.00137.
19. Vascellari, S.; Palmas, V.; Melis, M.; Pisanu, S.; Cusano, R.; Uva, P.; Perra, D.; Madau, V.; Sarchioto, M.; Oppo, V.; et al. Gut Microbiota and Metabolome Alterations Associated with Parkinson's Disease. *mSystems* **2020**, *5*, doi:10.1128/mSystems.00561-20.
20. Cosma-Grigorov, A.; Meixner, H.; Mrochen, A.; Wirtz, S.; Winkler, J.; Marxreiter, F. Changes in Gastrointestinal Microbiome Composition in PD: A Pivotal Role of Covariates. *Front Neurol* **2020**, *11*, 1041, doi:10.3389/fneur.2020.01041.
21. Nishiwaki, H.; Ito, M.; Ishida, T.; Hamaguchi, T.; Maeda, T.; Kashihara, K.; Tsuboi, Y.; Ueyama, J.; Shimamura, T.; Mori, H.; et al. Meta-Analysis of Gut Dysbiosis in Parkinson's Disease. *Mov Disord* **2020**, *35*, 1626–1635, doi:10.1002/mds.28119.
22. Cirstea, M.S.; Yu, A.C.; Golz, E.; Sundvick, K.; Kliger, D.; Radisavljevic, N.; Foulger, L.H.; Mackenzie, M.; Huan, T.; Finlay, B.B.; et al. Microbiota Composition and Metabolism Are Associated With Gut Function in Parkinson's Disease. *Mov Disord* **2020**, *35*, 1208–1217, doi:10.1002/mds.28052.
23. Vogt, N.M.; Kerby, R.L.; Dill-McFarland, K.A.; Harding, S.J.; Merluzzi, A.P.; Johnson, S.C.; Carlsson, C.M.; Asthana, S.; Zetterberg, H.; Blennow, K.; et al. Gut Microbiome Alterations in Alzheimer's Disease. *Sci Rep* **2017**, *7*, 13537, doi:10.1038/s41598-017-13601-y.
24. Cattaneo, A.; Cattane, N.; Galluzzi, S.; Provasi, S.; Lopizzo, N.; Festari, C.; Ferrari, C.; Guerra, U.P.; Paghera, B.; Muscio, C.; et al. Association of Brain Amyloidosis with Pro-Inflammatory Gut Bacterial Taxa and Peripheral Inflammation Markers in Cognitively Impaired Elderly. *Neurobiol. Aging* **2017**, *49*, 60–68, doi:10.1016/j.neurobiolaging.2016.08.019.
25. Zhuang, Z.-Q.; Shen, L.-L.; Li, W.-W.; Fu, X.; Zeng, F.; Gui, L.; Lü, Y.; Cai, M.; Zhu, C.; Tan, Y.-L.; et al. Gut Microbiota Is Altered in Patients with Alzheimer's Disease. *J. Alzheimers Dis.* **2018**, *63*, 1337–1346, doi:10.3233/JAD-180176.
26. Haran, J.P.; Bhattarai, S.K.; Foley, S.E.; Dutta, P.; Ward, D.V.; Bucci, V.; McCormick, B.A. Alzheimer's Disease Microbiome Is Associated with Dysregulation of the Anti-Inflammatory P-Glycoprotein Pathway. *MBio* **2019**, *10*, doi:10.1128/mBio.00632-19.
27. Lopizzo, N.; Provasi, S.; Marizzoni, M.; Borruso, L.; Andryszak, P.; Frisoni, G.B.; Cattaneo, A. Identification of Gut Microbiota Signature in Alzheimer's Disease: Possible Role in Influencing Peripheral Inflammation. *European Neuropsychopharmacology* **2019**, *29*, S167–S168, doi:10.1016/j.euroneuro.2018.11.289.
28. Liu, P.; Wu, L.; Peng, G.; Han, Y.; Tang, R.; Ge, J.; Zhang, L.; Jia, L.; Yue, S.; Zhou, K.; et al. Altered Microbiomes Distinguish Alzheimer's Disease from Amnestic Mild Cognitive Impairment and Health in a Chinese Cohort. *Brain, Behavior, and Immunity* **2019**, *80*, 633–643, doi:10.1016/j.bbi.2019.05.008.
29. Li, B.; He, Y.; Ma, J.; Huang, P.; Du, J.; Cao, L.; Wang, Y.; Xiao, Q.; Tang, H.; Chen, S. Mild Cognitive Impairment Has Similar Alterations as Alzheimer's Disease in Gut Microbiota. *Alzheimer's & Dementia* **2019**, doi:10.1016/j.jalz.2019.07.002.
30. Miyake, S.; Kim, S.; Suda, W.; Oshima, K.; Nakamura, M.; Matsuoka, T.; Chihara, N.; Tomita, A.; Sato, W.; Kim, S.-W.; et al. Dysbiosis in the Gut Microbiota of Patients with Multiple Sclerosis, with a Striking Depletion of Species Belonging to Clostridia XIVa and IV Clusters. *PLoS ONE* **2015**, *10*, e0137429, doi:10.1371/journal.pone.0137429.
31. Cantarel, B.L.; Waubant, E.; Chehoud, C.; Kuczynski, J.; DeSantis, T.Z.; Warrington, J.; Venkatesan, A.; Fraser, C.M.; Mowry, E.M. Gut Microbiota in Multiple Sclerosis: Possible Influence of Immunomodulators. *J. Investig. Med.* **2015**, *63*, 729–734, doi:10.1097/JIM.0000000000000192.
32. Chen, J.; Chia, N.; Kalari, K.R.; Yao, J.Z.; Novotna, M.; Paz Soldan, M.M.; Luckey, D.H.; Marietta, E.V.; Jeraldo, P.R.; Chen, X.; et al. Multiple Sclerosis Patients Have a Distinct Gut Microbiota Compared to Healthy Controls. *Sci Rep* **2016**, *6*, 28484, doi:10.1038/srep28484.
33. Tremlett, H.; Fadrosh, D.W.; Faruqi, A.A.; Zhu, F.; Hart, J.; Roalstad, S.; Graves, J.; Lynch, S.; Waubant, E.; US Network of Pediatric MS Centers Gut Microbiota in Early Pediatric Multiple Sclerosis: A Case-Control Study. *Eur. J. Neurol.* **2016**, *23*, 1308–1321, doi:10.1111/ene.13026.
34. Tremlett, H.; Waubant, E. The Gut Microbiota and Pediatric Multiple Sclerosis: Recent Findings. *Neurotherapeutics* **2018**, *15*, 102–108, doi:10.1007/s13311-017-0574-3.
35. Jangi, S.; Gandhi, R.; Cox, L.M.; Li, N.; von Glehn, F.; Yan, R.; Patel, B.; Mazzola, M.A.; Liu, S.; Glanz, B.L.; et al. Alterations of the Human Gut Microbiome in Multiple Sclerosis. *Nat Commun* **2016**, *7*, 12015, doi:10.1038/ncomms12015.

36. Cekanaviciute, E.; Yoo, B.B.; Runia, T.F.; Debelius, J.W.; Singh, S.; Nelson, C.A.; Kanner, R.; Bencosme, Y.; Lee, Y.K.; Hauser, S.L.; et al. Gut Bacteria from Multiple Sclerosis Patients Modulate Human T Cells and Exacerbate Symptoms in Mouse Models. *Proc. Natl. Acad. Sci. U.S.A.* **2017**, *114*, 10713–10718, doi:10.1073/pnas.1711235114.

37. Forbes, J.D.; Chen, C.; Knox, N.C.; Marrie, R.-A.; El-Gabalawy, H.; de Kievit, T.; Alfa, M.; Bernstein, C.N.; Van Domselaar, G. A Comparative Study of the Gut Microbiota in Immune-Mediated Inflammatory Diseases—Does a Common Dysbiosis Exist? *Microbiome* **2018**, *6*, doi:10.1186/s40168-018-0603-4.

38. Kozhieva, M.; Naumova, N.; Alikina, T.; Boyko, A.; Vlassov, V.; Kabilov, M.R. Primary Progressive Multiple Sclerosis in a Russian Cohort: Relationship with Gut Bacterial Diversity. *BMC Microbiol* **2019**, *19*, doi:10.1186/s12866-019-1685-2.

39. Ventura, R.E.; Iizumi, T.; Battaglia, T.; Liu, M.; Perez-Perez, G.I.; Herbert, J.; Blaser, M.J. Gut Microbiome of Treatment-Naïve MS Patients of Different Ethnicities Early in Disease Course. *Scientific Reports* **2019**, *9*, 16396, doi:10.1038/s41598-019-52894-z.

40. Oezguen, N.; Yalcinkaya, N.; Kücükali, C.I.; Dahdouli, M.; Hollister, E.B.; Luna, R.A.; Türkoglu, R.; Kürtüncü, M.; Eraksoy, M.; Savidge, T.C.; et al. Microbiota Stratification Identifies Disease-Specific Alterations in Neuro-Behçet's Disease and Multiple Sclerosis. *Clin. Exp. Rheumatol.* **2019**, *37 Suppl 121*, 58–66.

41. Yin, J.; Liao, S.-X.; He, Y.; Wang, S.; Xia, G.-H.; Liu, F.-T.; Zhu, J.-J.; You, C.; Chen, Q.; Zhou, L.; et al. Dysbiosis of Gut Microbiota With Reduced Trimethylamine-N-Oxide Level in Patients With Large-Artery Atherosclerotic Stroke or Transient Ischemic Attack. *J Am Heart Assoc* **2015**, *4*, doi:10.1161/JAHA.115.002699.

42. Yamashiro, K.; Tanaka, R.; Urabe, T.; Ueno, Y.; Yamashiro, Y.; Nomoto, K.; Takahashi, T.; Tsuji, H.; Asahara, T.; Hattori, N. Gut Dysbiosis Is Associated with Metabolism and Systemic Inflammation in Patients with Ischemic Stroke. *PLoS ONE* **2017**, *12*, e0171521, doi:10.1371/journal.pone.0171521.

43. Xia, G.-H.; You, C.; Gao, X.-X.; Zeng, X.-L.; Zhu, J.-J.; Xu, K.-Y.; Tan, C.-H.; Xu, R.-T.; Wu, Q.-H.; Zhou, H.-W.; et al. Stroke Dysbiosis Index (SDI) in Gut Microbiome Are Associated With Brain Injury and Prognosis of Stroke. *Front Neurol* **2019**, *10*, 397, doi:10.3389/fneur.2019.00397.

44. Li, N.; Wang, X.; Sun, C.; Wu, X.; Lu, M.; Si, Y.; Ye, X.; Wang, T.; Yu, X.; Zhao, X.; et al. Change of Intestinal Microbiota in Cerebral Ischemic Stroke Patients. *BMC Microbiol.* **2019**, *19*, 191, doi:10.1186/s12866-019-1552-1.

45. Li, H.; Zhang, X.; Pan, D.; Liu, Y.; Yan, X.; Tang, Y.; Tao, M.; Gong, L.; Zhang, T.; Woods, C.R.; et al. Dysbiosis Characteristics of Gut Microbiota in Cerebral Infarction Patients. *Transl Neurosci* **2020**, *11*, 124–133, doi:10.1515/tnsci-2020-0117.

46. Haak, B.W.; Westendorp, W.F.; van Engelen, T.S.R.; Brands, X.; Brouwer, M.C.; Vermeij, J.-D.; Hugenholtz, F.; Verhoeven, A.; Derks, R.J.; Giera, M.; et al. Disruptions of Anaerobic Gut Bacteria Are Associated with Stroke and Post-Stroke Infection: A Prospective Case-Control Study. *Transl Stroke Res* **2020**, doi:10.1007/s12975-020-00863-4.

47. Fang, X.; Wang, X.; Yang, S.; Meng, F.; Wang, X.; Wei, H.; Chen, T. Evaluation of the Microbial Diversity in Amyotrophic Lateral Sclerosis Using High-Throughput Sequencing. *Front Microbiol* **2016**, *7*, doi:10.3389/fmicb.2016.01479.

48. Brenner, D.; Hiergeist, A.; Adis, C.; Mayer, B.; Gessner, A.; Ludolph, A.C.; Weishaupt, J.H. The Fecal Microbiome of ALS Patients. *Neurobiology of Aging* **2018**, *61*, 132–137, doi:10.1016/j.neurobiolaging.2017.09.023.

49. Mazzini, L.; Mogna, L.; De Marchi, F.; Amoruso, A.; Pane, M.; Aloisio, I.; Cionci, N.B.; Gaggìa, F.; Lucenti, A.; Bersano, E.; et al. Potential Role of Gut Microbiota in ALS Pathogenesis and Possible Novel Therapeutic Strategies. *J. Clin. Gastroenterol.* **2018**, *52 Suppl 1, Proceedings from the 9th Probiotics, Prebiotics and New Foods, Nutraceuticals and Botanicals for Nutrition&Human and Microbiota Health Meeting, held in Rome, Italy from September 10 to 12, 2017*, S68–S70, doi:10.1097/MCG.0000000000001042.

50. Zhai, C.-D.; Zheng, J.-J.; An, B.-C.; Huang, H.-F.; Tan, Z.-C. Intestinal Microbiota Composition in Patients with Amyotrophic Lateral Sclerosis: Establishment of Bacterial and Archaeal Communities Analyses. *Chin. Med. J.* **2019**, *132*, 1815–1822, doi:10.1097/CM9.0000000000000351.

51. Zeng, Q.; Shen, J.; Chen, K.; Zhou, J.; Liao, Q.; Lu, K.; Yuan, J.; Bi, F. The Alteration of Gut Microbiome and Metabolism in Amyotrophic Lateral Sclerosis Patients. *Sci Rep* **2020**, *10*, 12998, doi:10.1038/s41598-020-69845-8.

52. Di Gioia, D.; Bozzi Cionci, N.; Baffoni, L.; Amoruso, A.; Pane, M.; Mogna, L.; Gaggìa, F.; Lucenti, M.A.; Bersano, E.; Cantello, R.; et al. A Prospective Longitudinal Study on the Microbiota Composition in Amyotrophic Lateral Sclerosis. *BMC Med* **2020**, *18*, 153, doi:10.1186/s12916-020-01607-9.
